# *Staphylococcus aureus* specific lung resident memory CD4^+^ Th1 cells attenuate the severity of influenza virus induced secondary bacterial pneumonia

**DOI:** 10.1038/s41385-022-00529-4

**Published:** 2022-05-30

**Authors:** Jessica Braverman, Ian R. Monk, Chenghao Ge, Glen P. Westall, Timothy P. Stinear, Linda M. Wakim

**Affiliations:** 1 Department of Microbiology and Immunology, The University of Melbourne, at the Peter Doherty Institute for Infection and Immunity, Melbourne, VIC 3000 Australia; 2 School of Medicine, Tsinghua University, Beijing, China; 3 Lung Transplant Service, Alfred Hospital, Melbourne, Victoria, Australia; Department of Medicine, Monash University, Melbourne, VIC Australia

## Abstract

*Staphylococcus aureus* is a major cause of severe pulmonary infections. The evolution of multi-drug resistant strains limits antibiotic treatment options. To date, all candidate vaccines tested have failed, highlighting the need for an increased understanding of the immunological requirements for effective *S. aureus* immunity. Using an *S. aureus* strain engineered to express a trackable CD4^+^ T cell epitope and a murine model of *S. aureus* pneumonia, we show strategies that lodge Th1 polarised bacterium specific CD4^+^ tissue resident memory T cells (Trm) in the lung can significantly attenuate the severity of *S. aureus* pneumonia. This contrasts natural infection of mice that fails to lodge CD4^+^ Trm cells along the respiratory tract or provide protection against re-infection, despite initially generating Th17 bacterium specific CD4^+^ T cell responses. Interestingly, lack of CD4^+^ Trm formation after natural infection in mice appears to be reflected in humans, where the frequency of *S. aureus* reactive CD4^+^ Trm cells in lung tissue is also low. Our findings reveal the protective capacity of *S. aureus* specific respiratory tract CD4^+^ Th1 polarised Trm cells and highlight the potential for targeting these cells in vaccines that aim to prevent the development of *S. aureus* pneumonia.

## Introduction

*Staphylococcus aureus* is a Gram-positive bacterium, that persistently colonises up to 30% of the human population. Despite being able to exist as a benign human commensal, *S. aureus* is also a versatile human pathogen, causing a diverse array of life-threatening infections, including endocarditis, osteomyelitis, necrotizing pneumonia, and septic shock^1,2^. Pulmonary *S. aureus* infections are a substantial public health concern; they have a very high case fatality rate and are the leading cause of nosocomial pneumonia and secondary bacterial pneumonia following influenza A virus infection. The clinical management of staphylococcal lung infections is complicated by widespread antibiotic resistance present in this bacterium^3^, and as antibiotics are currently the only available therapy, treatment failures are becoming increasingly common (10–30% mortality rate)^4,5^. An effective vaccine against *S. aureus*, including drug-resistant strains, would save lives and offer a potential solution to the epidemic of antibiotic resistance.

To date, all efforts to develop an *S. aureus* vaccine have been unsuccessful. While antibody-based vaccines targeting polysaccharide or protein antigens are highly protective against diseases caused by many bacterial pathogens (including *Streptococcus pneumoniae*, *Haemophilus influenzae* type b, and *Neisseria meningitidis*^6,7^), this type of vaccine has so far proven to be ineffective against *S. aureus*^8^. The failure of antibody-based vaccines in the prevention of *S. aureus* infections has brought into question the immunological requirements for effective *S. aureus* defence. Evidence from both mouse and human studies support a role for memory CD4^+^ T cells in the development of protective immunity against invasive *S. aureus* infections^9–12^. Despite the growing evidence demonstrating that memory CD4^+^ T cells can contribute to protective immunity against *S. aureus* infections, the subset and polarity of the memory CD4^+^ T cell pool mediating this bacterial control, and whether these change if the infection is systemic or localised in nature, remains unclear.

Memory T cells can be segregated into three subsets: two circulating T cell subsets, termed central (Tcm) and effector memory T cells (Tem), and a noncirculating memory T cell pool that is resident within peripheral tissues (Trm). Resident memory T cells represent a population of self-sustaining memory T cells that are deposited in most organs (brain, intestine, female reproductive tract, salivary glands, lung, skin, and liver)^13–17^ and have been shown to be an essential component of immune defence against an array of pulmonary viral (influenza, RSV, SARs-CoV-1)^18–22^ and bacterial infections (*Streptococcus pneumoniae, Bordetella pertussis*, *Klebsiella pneumoniae*, *Mycobacterium tuberculosis*)^23–25^. Marked by the expression of CD69 and, in some cases CD103, these cells orchestrate local recall responses, rapidly gaining effector function^26,27^ and facilitating the accelerated recruitment of circulating immune cells to the site of infection^28–30^. Here, using an *S. aureus* strain we engineered to express a trackable CD4^+^ T cell epitope and a murine model of *S. aureus* pneumonia^31^, we set out to map the bacterium specific CD4^+^ T cell response and identify the subset and polarity of the memory CD4^+^ T cell pool capable of providing protection against *S. aureus* pneumonia. Our findings reveal the protective capacity *S. aureus* specific respiratory tract CD4^+^ Th1 polarised Trm cells and highlight the potential for targeting these cells in vaccines that aim to prevent the development of *S. aureus* pneumonia.

## Results

### Recombinant *S. aureus* engineered to express HSV-1 glycoprotein D (gD) epitope activates gDT-2 CD4^+^ TCR transgenic T cells

**Fig. 1:**
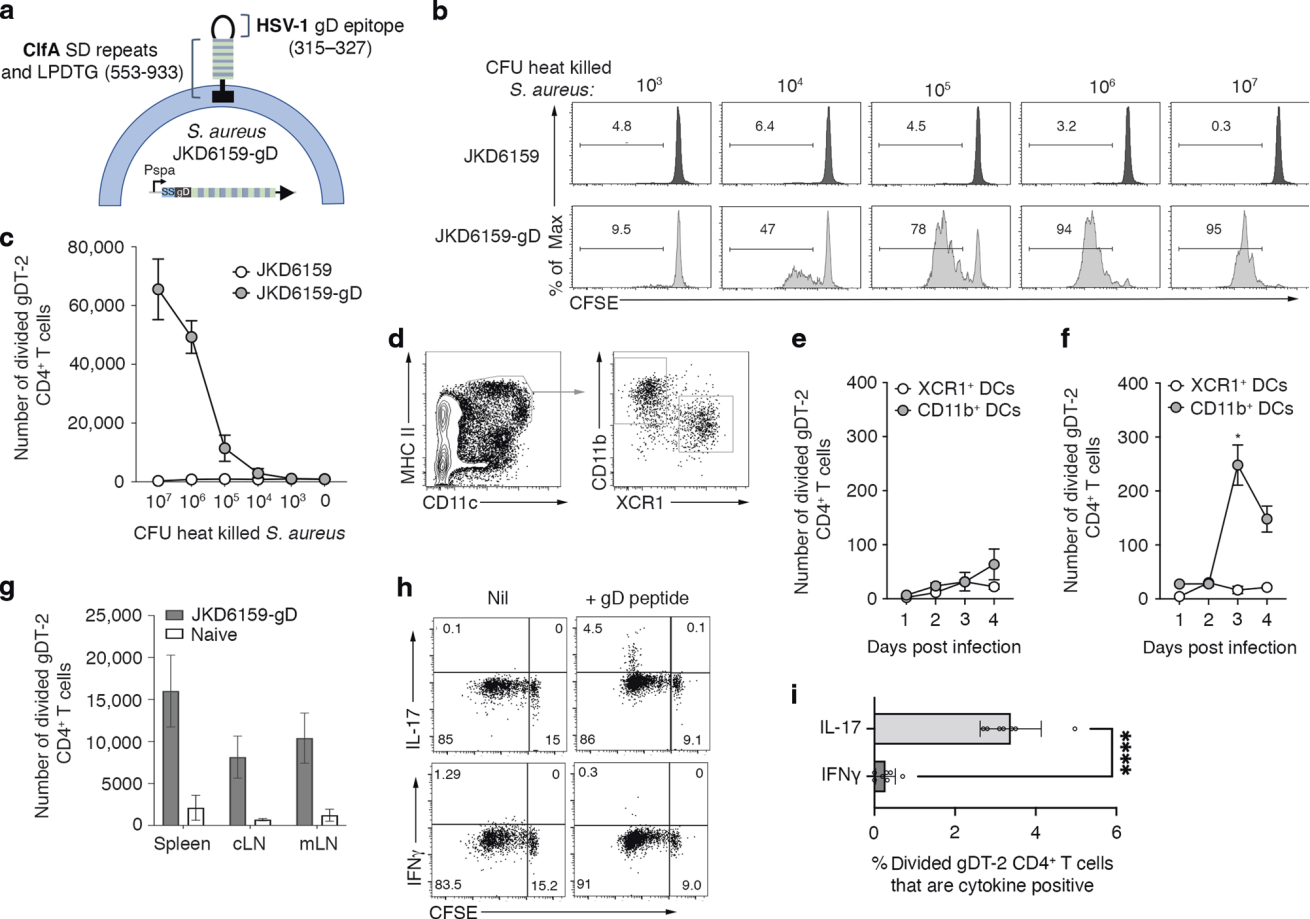
Recombinant *S. aureus* engineered to express HSV-1 glycoprotein D (gD) activates gDT-2 CD4^+^ TCR transgenic T cells. **a** Schematic of the JKD6159-gD bacteria. A fusion protein (gD-SD) was expressed from the Protein A promoter (P*spa*) and secreted from the *S. aureus* cell via the N-terminal Protein A signal sequence (SS). gD-SD comprises the HSV-1 glycoprotein D epitope (amino acids 315–327) fused to the serine – aspartic acid repeats and LPDTG sortase A motif (cell wall cross linked) from the surface protein ClfA (amino acids 553–933). The construct was integrated into the *attB* site between the *isdB – rpmF* genes on the chromosome of JKD6159, yielding strain JKD6159-gD. **b**, **c** JKD6159-gD activates gDT-2 CD4^*+*^ *T cells* in vitro. 5 × 10^4^ CFSE labelled gDT-2 CD45.1^+^ CD4^+^ T cells and 1 × 10^4^ splenic dendritic cells were cultured with varying doses (10^3^-10^7^ CFU) of heat-killed *S. aureus* (parental JKD6159 or the recombinant JKD6159-gD strain engineered to express the gD epitope from HSV-1) and the proportion of divided gDT-2 CD4^+^ T cells was measured 60 h later. **b** Representative flow cytometry profiles of gDT-2 CD4^+^ T cells showing levels of CFSE. **c** The graph depicts the absolute number of divided (CFSE^lo^) gDT-2 CD4^+^ T cells. Symbols represent the mean ± SEM. Data representative of 3 independent experiments. **d**–**f** CD11b^+^ DCs in the mLN present bacterial antigen following intranasal JKD6159-gD infection. Proliferation of CFSE-labelled gDT-2 CD4^+^ T cells cultured for 60 h together with CD11b^+^ and XCR1^+^ DCs (identified as shown in **d**) isolated from the (**e**) cLN or (**f**) mLN of mice that received an intranasal total respiratory tract (TRT) infection 1–4 days earlier with 10^8^ CFU of JKD6159-gD. Data pooled from three experiments per time point. Symbols represent means ± SEM (two-way ANOVA, Sidak’s multiple comparison). **g**–**i** Intranasal infection with JKD6159-gD primes CD4^+^ Th17 cells. C57BL/6 (CD45.2) mice injected with 1 × 10^6^ CFSE labelled naïve gDT-2 CD45.1^+^ CD4^+^ T cells and that received an intranasal infection (TRT) with 10^8^ CFU JKD6159-gD were sacrificed 5 days later and the absolute number of divided gDT-2 CD4^+^ T cells (CD4^+^Va3.2^+^CD45.1^+^CFSE^lo^) in the mLN, cLN and spleen was measured. Bars represent the mean ± SEM (*n* = 5). The result is a combination of 2 independent experiments (**h**) Representative flow cytometry profiles gated on gDT-2 CD4^+^ T cells isolated from the mLN showing intracellular levels of IL-17 and IFNγ following a brief in vitro stimulation. **i** The proportion of CD4^+^ gDT-2 T cells in the mLN generating IFNγ or IL-17. Bars represent the mean ± SEM (*n* = 7 mice per group). The result is a combination of two independent experiments (student *t* test).

To explore the role of CD4^+^ T cell immunity in the control of *S. aureus* infection we generated a recombinant *S. aureus* strain that expresses a CD4^+^ T cell epitope derived from herpes simplex virus (HSV) glycoprotein D (gD_315-327_) (Fig. 1a) and tracked the CD4^+^ T cell response using T cell receptor (TCR) transgenic CD4^+^ gDT-2 cells which are specific for class II–restricted HSV gD^32^. This approach was necessary as endogenous *S. aureus* CD4^+^ T cell epitopes are currently undefined. We firstly confirmed that the gD expressing *S. aureus*, referred to hereafter as, JKD6159-gD could trigger gDT-2 CD4^+^ T cell activation, as measured by an in vitro antigen presentation assay. Splenic dendritic cells (DCs) and carboxyfluorescein succinimidyl ester (CFSE) labelled gDT-2 CD4^+^ T cells were cultured together with varying doses of heat killed JKD6159-gD, or as a control, the parental JKD6159 strain. The expansion of the gDT-2 CD4^+^ T cells was assessed 60 h post culture by the dilution of the CFSE dye. As shown in Fig. 1b, c, gDT-2 CD4^+^ T cells divided in a dose dependent manner when cultured with JKD6159-gD bacteria, and importantly, these transgenic T cells remained undivided following exposure to the parental JKD5159 strain. Next, we checked whether DCs sort-purified from the cervical (cLN) and mediastinal (mLN) lymph nodes, which drain the upper and lower respiratory tract respectively, recovered from mice infected intranasally 1–4 days prior with JKD6159-gD, could stimulate CD4^+^ gDT-2 cell proliferation directly ex vivo. The CD11b^+^ DC subset isolated from the mLN on days 3–4 post JKD6159-gD infection activated gDT-2 CD4^+^ T cells directly ex vivo (Fig. 1d–f). This observation is in line with other studies demonstrating that CD11b^+^ (cDC2) DCs perform most of the class-II presentation^33,34^. To examine whether JKD6159-gD could activate gDT-2 CD4^+^ T cells in vivo, CFSE labelled congenically marked (CD45.1^+^) gDT-2 T cells were transferred into C57BL/6 (CD45.2) mice prior to intranasal JKD6159-gD infection. By day 5 p.i. sizable populations of divided gDT-2 CD4^+^ T cells (CFSE^lo^) were detected in the spleen, mLN and cLN (Fig. 1g) and these cells where largely IL-17 producing Th17 (Fig. 1h, i).

### Intranasal infection with *S. aureus* activates bacterium specific CD4^+^ effector T cells that fail to convert into respiratory tract tissue resident CD4^+^ T cell memory

**Fig. 2:**
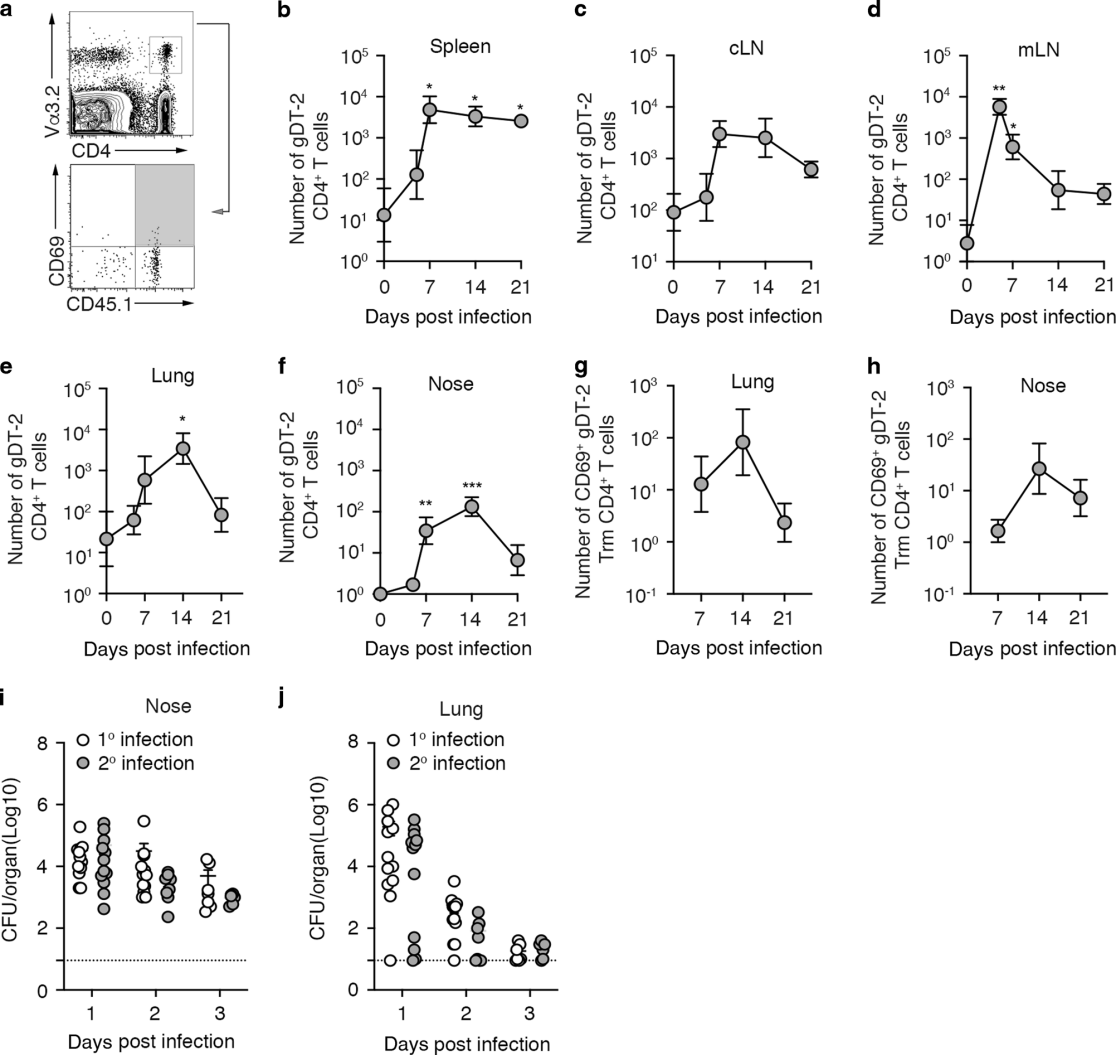
Intranasal infection with *S. aureus* activates bacterium specific CD4^+^ effector T cells that fail to convert into tissue resident CD4^+^ T cell memory. **a**–**h** Intranasal infection with JKD6159-gD generates poor CD4^+^ T cell memory. C57BL/6 (CD45.2) mice injected with 5 × 10^4^ naïve gDT-2 CD45.1^+^ CD4^+^ T cells and that received an intranasal infection (TRT) with 10^8^ CFU JKD6159-gD were sacrificed at days 5, 7, 14, and 21 post infection. **a** Representative flow cytometry profiles depict the gating strategy to identify gDT-2 CD4^+^ T cells (CD4^+^Va3.2^+^CD45.1^+^) and gDT-2 CD4^+^ tissue resident memory (Trm) (CD4^+^Va3.2^+^CD45.1^+^CD69^+^). The absolute number of total gDT-2 CD4^+^ T cells in the (**b**) spleen (**c**) cLN (**d**) mLN (**e**) lung, and (**f**) nose and the absolute number of gDT-2 CD4^+^ Trm in the (**g**) lung and (**h**) nose was measured. Symbols represent the mean ± SEM (*n* = 6 mice per time point). The result is a combination of two independent experiments (two-way ANOVA, Sidak’s multiple comparison). **i**–**j** Primary infection with *S. aureus* does not provide protection against secondary *S. aureus* challenge. Naïve C57BL/6 mice (1^o^ infection) or C57BL/6 mice infected intranasally (TRT) 21 days earlier with 10^8^ CFU of JKD6159 (2^o^ infection) were intranasally infected (TRT) with 10^8^ CFU of JKD6159 and at days 1–3 post infection the bacterial load in the (**i**) nose and (**j**) lung was measured. Symbols represent individual mice (*n* = 7–12 mice per timepoint) and the bars represent the mean ± SEM. The result is a combination of four independent experiments (two-way ANOVA, Sidak’s multiple comparison). Dotted line indicates the limit of detection.

To track the *S. aureus* specific CD4^+^ T cell response mounted following a pulmonary infection, gDT-2 CD45.1^+^ T cells were transferred into C57BL/6 recipient mice (CD45.2^+^) prior to an intranasal infection with JKD6159-gD and the number of gDT-2 CD4^+^ T cells (CD4^+^Va3.2^+^CD45.1^+^) in both lymphoid and respiratory tissue was measured over time. Intranasal infection with JKD6159-gD resulted in gDT-2 CD4^+^ T cell activation predominately within the mLN, these cells where then released into the circulation and by day 7 p.i gDT-2 CD4^+^ T cells were detectable in both the lung and nasal tissue (Fig. 2a–f). gDT-2 CD4^+^ T cells in the nasal tissue and lung continued to accumulate up until day 14 p.i., at which point numbers of gDT-2 cells in the upper and lower respiratory tract declined, and by day 21 p.i very few gDT-2 CD4^+^ T cell remained within these tissue compartments (Fig. 2e, f). Subset analysis of the gDT-2 cells in the lung and nasal tissue revealed that a fraction did express the resident memory T cell marker CD69, but these CD69^+^ gDT-2 CD4^+^ T cells were not retained within these tissues (Fig. 2g, h). Moreover, a secondary intranasal boost with JKD6159-gD did not significantly improve the size of the memory T cell pool in the circulation or the tissue compartments (Fig S1). These findings are in line with previous studies that show that *S. aureus* infection evokes poor memory T cell responses^35^.

Next, we checked whether this impairment in the development of a sizable bacterium specific memory CD4^+^ T cell pool left animals vulnerable to reinfection. Mice infected intranasally with 10^8^ CFU of *S. aureus* JKD6159 were rested for 21 days and then reinfected via the same route with the same dose of JKD6159 and the bacterial loads in the nose and lung were measured (Fig. 2I, j). The bacterial loads in the nose and lung tissue of re-infected animals matched the bacterial loads present in animals undergoing a primary infection, which suggests a primary infection with *S. aureus* does not induce protective immunity. Collectively, these data show that primary infection with *S. aureus* fails to evoke protective immunity and this correlates with an impairment in the persistence of *S. aureus* specific memory CD4^+^ T cells along the airways.

### Bacterium specific memory CD4^+^ T cells attenuate *S. aureus* pneumonia

**Fig. 3:**
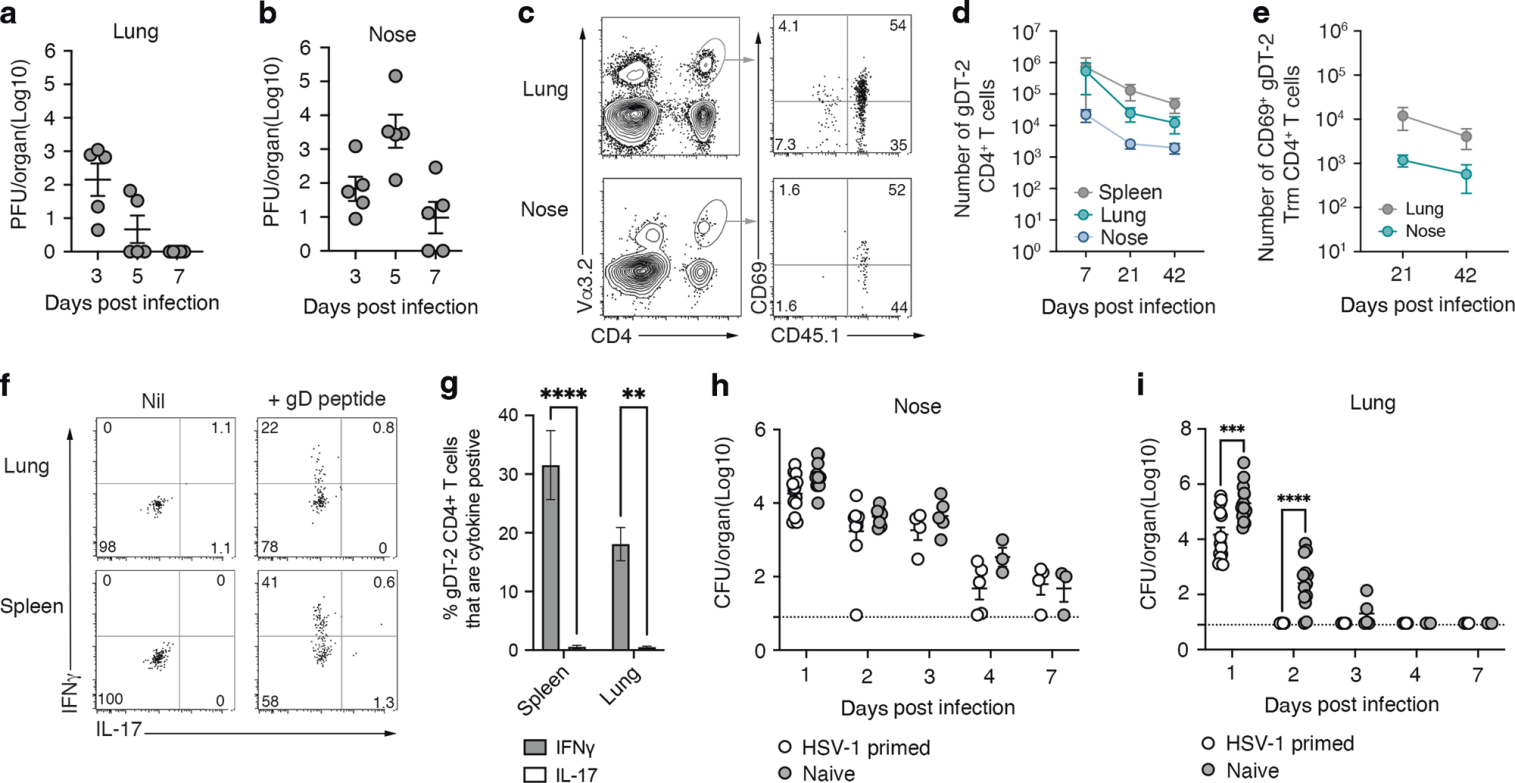
Bacterium specific memory CD4^+^ T cells attenuate *S. aureus* pneumonia. **a**, **b** Intranasal HSV-1 infection results in acute infection of the upper and lower airways. C57BL/6 mice received an intranasal infection (TRT) with 10^6^ PFU HSV-1 (SC16) and at the indicated days post infection the viral load in the nose and lung was measured. Symbols represent individual mice (*n* = 5 mice per timepoint) and the bars represent the mean ± SEM. The result is a combination of two independent experiments (**c**–**g**) gDT-2 CD4 Trm are deposited in the lung and nose following intranasal HSV-1 infection. C57BL/6 (CD45.2) mice injected with 5 × 10^4^ naïve gDT-2 CD45.1^+^ CD4^+^ T cells and that received an intranasal (TRT) infection with 10^6^ PFU HSV-1 (SC16) were sacrificed at day 7, 21, and 42 post infection. **c** Representative flow cytometry profiles at day 21 p.i. depict the gating strategy to identify gDT-2 CD4^+^ Trm (CD4^+^Va3.2^+^CD45.1^+^CD69^+^) (**d**) The absolute number of total gDT-2 CD4^+^ T cells (CD4^+^Va3.2^+^CD45.1^+^) in the spleen, lung and nose and (**e**) the absolute number of gDT-2.CD4^+^ Trm (CD4^+^Va3.2^+^CD45.1^+^CD69^+^) in the lung and nose was measured. Symbols represent the mean ± SEM (*n* = 5–7 mice per time point). The result is a combination of 4 independent experiments. **f** Representative flow cytometry profiles gated on gDT-2 CD4^+^ T cells isolated from the lung and spleen showing intracellular levels of IL-17 and IFNγ following a brief in vitro stimulation with gD peptide. **g** The proportion of CD4^+^ gDT-2 T cells in the spleen or lung generating IFNγ or IL-17. Bars represent the mean ± SEM (*n* = 4–5 mice per group). The result is a combination of two independent experiments (one-way ANOVA, Sidak’s multiple comparison). **h**, **i** Memory CD4^*+*^ gDT-2 cells are protective against pulmonary S. aureus infection. Naïve C57BL/6 mice (Naive) or C57BL/6 mice infected intranasally (TRT) 21 days earlier with 10^6^ PFU of HSV-1 were infected intranasally (TRT) with 10^8^ CFU of JKD6159-gD and at days 1–7 post infection the bacterial load in the (**h**) nose and (**i**) lung was measured. Symbols represent individual mice (*n* = 5–9 mice per timepoint) and the bars represent the mean ± SEM. The result is a combination of two independent experiments (student *t* test).

We next asked, if present in significant quantities, can *S. aureus* specific CD4^+^ memory T cells attenuate a pulmonary bacterial infection. To generate animals with a sizable pool of both circulating and respiratory tract resident *S. aureus* reactive CD4^+^ memory T cells, we seeded mice with gDT-2 CD45.1^+^ CD4^+^ T cells and infected them intranasally with HSV-1; this virus endogenously expresses the gD_315-327_ epitope and thus will trigger the activation of CD4^+^ gDT-2 cells. Intranasal infection with HSV-1 resulted in an acute viral infection of the airways, and infectious virus is cleared from this tissue by ~7 days post infection (Fig. 3a, b). Following the resolution of the pulmonary HSV-1 infection large populations of effector gDT-2 CD4^+^ T cells infiltrated the lung and nasal tissue and at early (day 21 p.i.) and late (day 42 p.i.) memory time points, gDT-2 CD4^+^ T cells were still detectable in the spleen, nose and lung (Fig. 3c, d). Subset analysis of the memory CD4^+^ T cells in the lung and nose revealed that the majority of memory CD4^+^ gDT-2 cells retained in these tissues expressed the CD69 Trm marker (Fig. 3e), were CD44^hi^ but CD103^lo^, were located in the parenchyma (Fig S2a) and, following a brief in vitro stimulation, synthesized IFNγ (Fig. 3f, g). To test whether these memory gDT-2 CD4^+^ T cells could confer protection against JKD6159-gD infection, naïve mice, or mice primed 21 days earlier with HSV-1, were challenged intranasally with JKD6159-gD and bacterial loads in the lung and nasal tissue were measured from days 1–7 p.i. While we observed no difference in bacterial loads or clearance rate of *S. aureus* in the nasal tissue between the two cohorts, the HSV-1 primed group more rapidly eradicated *S. aureus* from the lung (Fig. 3h, i) and this coincided with a rapid influx of CD4^+^ memory T cells into the airways (Fig S2b). On day 1 p.i. 10-fold less bacteria were detected in the lungs of HSV-1 primed mice compared to naïve controls. By day 2 p.i. all HSV-1 primed mice had eliminated the bacteria from the lung, while 84% of naïve mice still contained *S. aureus* in the lung at this time point (Fig. 3i). This enhanced clearance of bacteria from the lung tissue was antigen specific, as when HSV-1 primed mice were challenged with the parental *S. aureus* strain, which lacks the gD epitope, no attenuation in lung bacterial load was observed (Fig S2c, d). Moreover, when HSV-1 primed mice were challenged with a high dose of JKD6159-gD (4 × 10^8^ CFU), which results in 50% of naïve animals succumbing to infection within 24 h of inoculation, all of the HSV-1 primed animals survived the challenge (Fig S2e). These data show that *S. aureus* reactive CD4^+^ memory T cells facilitate the rapid clearance of the bacteria from the lung.

### Differences in T cell homing molecule expression, polarity and pulmonary inflammation following infection with HSV-1 and *S. aureus*

**Fig. 4:**
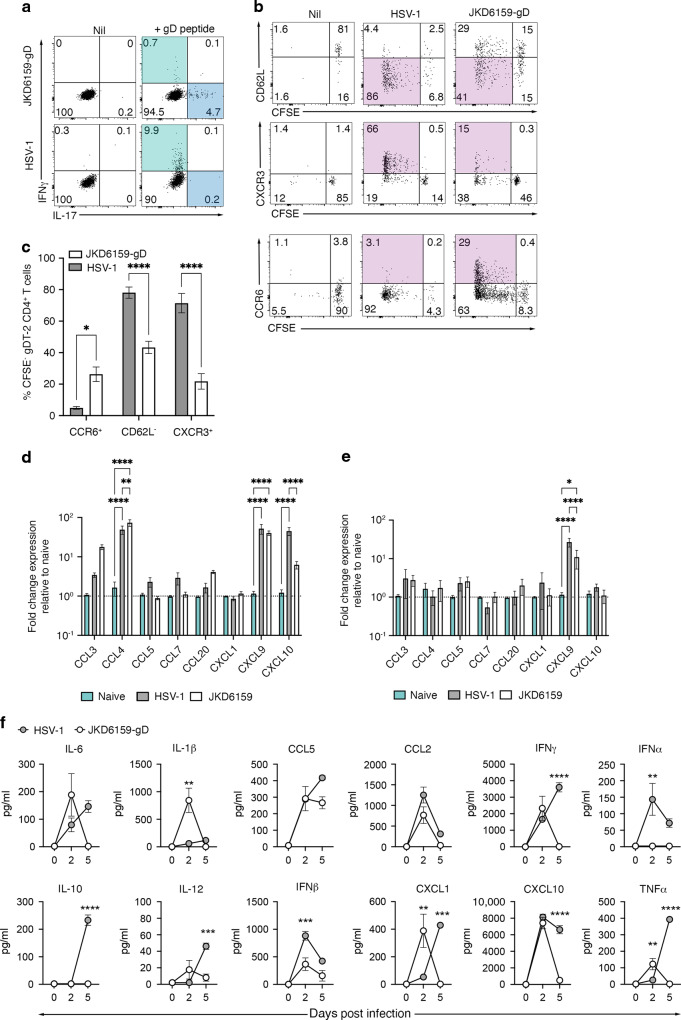
Lung inflammatory profile and CD4^+^ T cell polarity evoked following pulmonary *S. aureu*s and HSV-1 infection. C57BL/6 (CD45.2) mice injected with 1 × 10^6^ CFSE labelled naïve gDT-2 CD45.1^+^ CD4^+^ T cells and that received an intranasal infection (TRT) with either 10^8^ CFU JKD6159-gD or 10^6^ PFU HSV-1 were sacrificed 4 days later and the proportion of divided gDT-2 CD4^+^ T cells (CD4^+^Va3.2^+^CD45.1^+^CFSE^lo^) in the mLN expressing CD62L, CXCR3, and CCR6 was measured. **a** Representative flow cytometry profiles gated on gDT-2 CD4^+^ T cells isolated from the mLN showing intracellular levels of IL-17 and IFNγ following a brief in vitro stimulation. **b** Representative flow cytometry profiles gated on gDT-2 CD4^+^ T cells isolated from the mLN showing the level of expression of the indicated markers (**c**) Graph shows the proportion of divided cells that are CCR6^+^, CD62L^−^, or CXCR3^+^. Bars represent the mean ± SEM (*n* = 6–7). The results are a combination of three independent experiments (two-way ANOVA, Sidak’s multiple comparison). **d**, **e** C57BL/6 mice were intranasally infected (TRT) with 10^8^ CFU of *S. aureus* (JKD6159-gD) or 10^6^ PFU HSV-1 and (**d**) 2 days and (**e**) 5 days later the expression level of a panel of chemokines in lung tissue homogenates was measured by RT-PCR. Bars represent the mean fold change in expression relative to naïve lung tissue ± SEM (*n* = 5 mice per group). Data are pooled from two independent experiments (two-way ANOVA, Tukey’s multiple comparison). **f** C57BL/6 mice were intranasally infected (TRT) with 10^8^ CFU of *S. aureus* (JKD6159-gD) or 10^6^ PFU HSV-1 and 2 or 5 days later the level of a panel of cytokines/chemokines in lung tissue homogenates was measured by cytometric bead array. Symbols represent the mean ± SEM (*n* = 5 mice per group). Data are pooled from 2 independent experiments (two-way ANOVA, Sidak’s multiple comparison).

Intranasal infection with HSV-1 results in the recruitment of a sizable pool of effector gDT-2 CD4^+^ T cells into the lung and nasal tissue and the retention of gDT-2 CD4^+^ Trm along the respiratory tract. In contrast, the delivery of *S. aureus* JKD6159-gD via the same route resulted in poor seeding and retention of gDT-2 CD4^+^ Trm in the lung and nasal tissue. We next explored whether these differences in CD4^+^ T cell recruitment and retention following infection with these two pathogens could be explained by the development of CD4^+^ T cells with different polarities and/or the expression of different chemokine receptors required for T cell trafficking into the respiratory tissue. Mice seeded with CFSE labelled gDT-2 CD45.1^+^ T cells were infected intranasally with either 10^6^ PFU of HSV-1 or 10^8^ CFU of JKD6159-gD. On day 4 p.i., the mLN were recovered and the divided gDT-2 CD4^+^T cells (CFSE^lo^) were assessed for intracellular IFNγ and IL-17 levels, and the expression of a panel of chemokine receptors/homing molecules known to facilitate T cell trafficking to the lung including, CD62L, CXCR3, and CCR6^36^. Infection with HSV-1 resulted in the generation of predominately IFNγ producing Th1 cells, and assessment of the divided cells (CFSE^lo^) revealed that 78% downregulated CD62L, 5% upregulated CCR6, while 70% upregulated CXCR3 (Fig. 4a–c). In contrast, following infection with JKD6159-gD, activated CD4^+^ T cells were predominantly IL-17 producing Th17, and of the divided cells, 40% downregulated CD62L, 26% upregulated CCR6, while only 20% upregulated CXCR3 (Fig. 4a–c). Hence infection with *S. aureus* and HSV-1 triggers the activation of CD4^+^ T cells with different polarities and varying levels of expression of homing receptors linked to migration into inflamed lung tissue^36^. An assessment of the expression of a panel of chemokines in lung tissue homogenates of mice on days 2 and 5 post infection revealed that while both *S. aureus* and HSV-1 infection triggered the expression of CCL4, CXCL9, and CXCL10, by day 5 p.i., only CXCL9, which elicits its chemotactic function by interacting with CXCR3, was still elevated in the lung tissue in both infection models (Fig. 4d, e). We also measured the levels of a panel of inflammatory cytokines/chemokines in lung tissue homogenates by cytometric bead array and observed that while both pathogens initially triggered the production of most of the inflammatory cytokines/chemokines tested, by day 5 p.i., inflammation was largely resolved in the *S. aureus* infected mice, while several pro-inflammatory molecules (IFNγ, IL-10, IL-12, CXCL1, CXCL10, TNFα) remained significantly elevated in the HSV-1 infected lung tissue (Fig. 4f). These data show that intranasal *S. aureus* infection evokes transient pulmonary inflammation and the generation of effector CD4^+^ T cells that express chemokine receptors which do not match the chemotactic profile generated in the *S. aureus* infected lung tissue. Collectively, this may in part explain the poor recruitment and ultimate retention of *S. aureus* specific CD4^+^ T cells in the lung following bacterial infection.

### Bacterium specific respiratory tract CD4^+^ Trm limit the severity of influenza virus induced *S. aureus* pneumonia

**Fig. 5:**
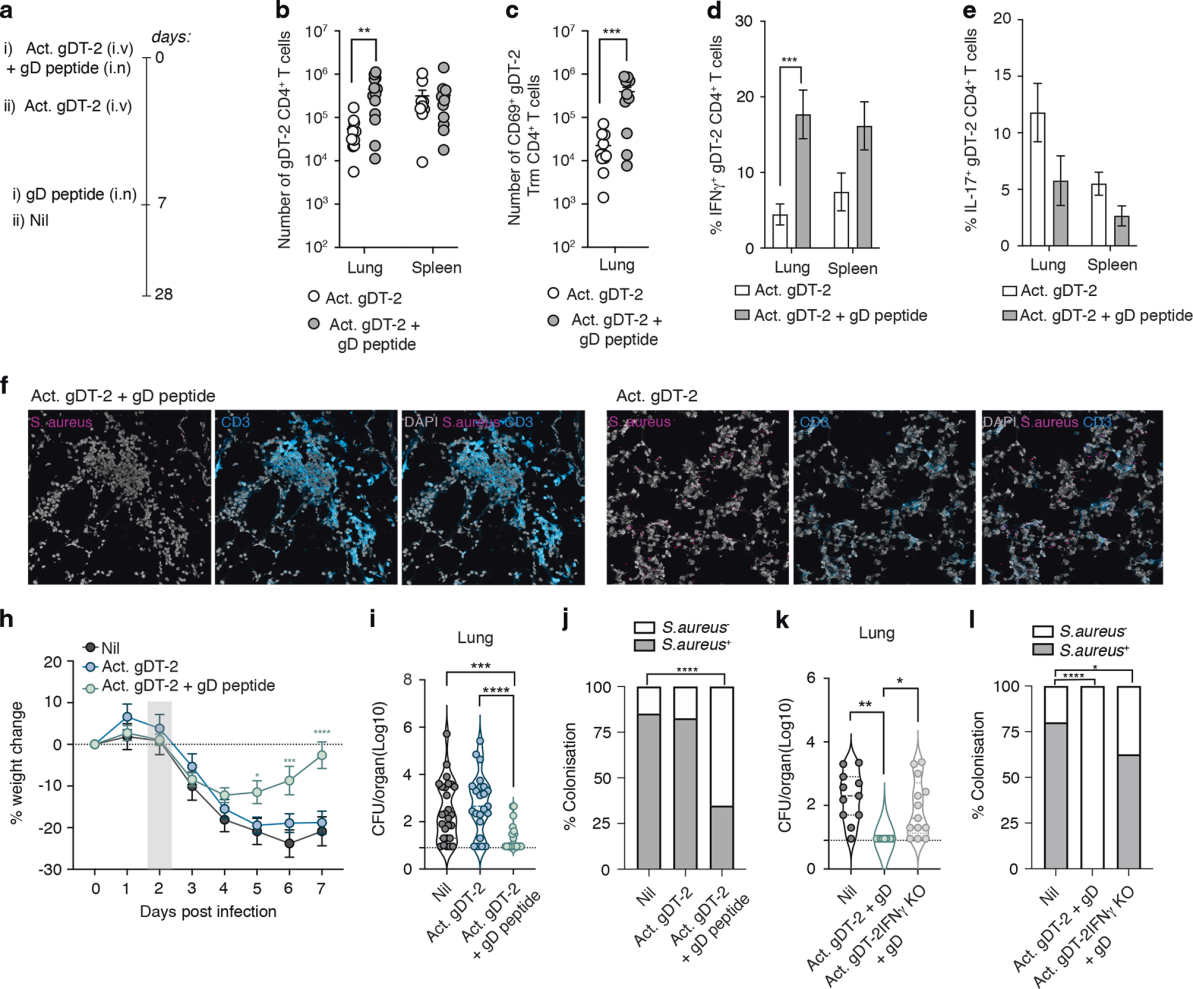
Bacteria specific respiratory tract CD4^+^ Th1 polarised Trm limit the severity of influenza virus induced *S. aureus* pneumonia. **a**–**e** Intranasal immunisation with gD peptide generates respiratory tract gDT-2 CD4^*+*^ Trm. C57BL/6 (CD45.2) mice injected with 5 × 10^6^ in vitro activated gDT-2 CD45.1^+^ CD4^+^ T cells were inoculated at day 0 and day 7 via the intranasal route with 1 μg LPS with or without 30 μg gD peptide. **a** Schematic of the experimental timeline. At day 28 post immunisation the (**b**) absolute number of total gDT-2 CD4^+^ T cells (CD4^+^Va3.2^+^CD45.1^+^) in the spleen and lung and (**c**) the absolute number of gDT-2 CD4^+^ Trm (CD4^+^Va3.2^+^CD45.1^+^CD69^+^) in the lung was measured. Symbols represent individual mice, and the line represents the mean ± SEM (*n* = 10–13 mice per group). The result is a combination of four independent experiments (two-way ANOVA, Sidak’s multiple comparison). **d**, **e** The proportion of CD4^+^ gDT-2 T cells in the spleen and lung generating (**d**) IFNγ and (**e**) IL-17 following a brief in vitro stimulation. Bars represent the mean ± SEM (*n* = 9 mice per group). The result is a combination of three independent experiments (two-way ANOVA, Sidak’s multiple comparison). **f**–**j** Lodging bacterium specific CD4^*+*^ Trm along the respiratory tract limits the severity of influenza induced S. aureus pneumonia. Mice generated as described in **a** and a cohort of naïve animals (Nil) were intranasally infected (TRT) with 10^4^ PFU influenza virus (X31, H3N2) and 2 days later, were infected intranasally (TRT) with 10^8^ CFU of JKD6159-gD. **f**, **g** Fluorescent microscopy (x20 magnification) on lung tissue sections of mice on days 2 post *S. aureus* infection with CD3 (blue), *S. aureus* (purple) and DAPI staining. **h** The graph depicts the percentage weight change measured over the course of the experiment. The symbols represent the mean ± SEM (*n* = 8–9 mice per group). The grey band indicates the day on which *S. aureus* was administered. The result is a combination of three independent experiments (two-way ANOVA, Dunnett’s multiple comparison) (**i**, **j**) The bacterial load in the (**i**) lung was measured on day 5 post *S. aureus* infection. Symbols represent individual mice (*n* = 20–24 mice per group). The result is a combination of seven independent experiments (one-way ANOVA, Tukey’s multiple comparison). The dotted line represents the limit of detection. **j** Graph depicts the proportion of mice that were colonised at day 5 post bacterial infection (Fisher’s exact test). **k**, **l** IFNγ production by bacterium specific CD4^*+*^ Trm is required to limit disease severity of influenza induced S. aureus pneumonia. C57BL/6 (CD45.2) mice injected with 5 × 10^6^ in vitro activated gDT-2 CD45.1^+^ CD4^+^ T cells or gDT-2 CD45.1^+^IFNγ KO CD4^+^ T cells were inoculated at day 0 and day 7 via the intranasal route with 1 μg LPS with 30 μg gD peptide, rested for 28 days and infected intranasally with 10^4^ PFU influenza virus (X31, H3N2) and 2 days later, were infected intranasally (TRT) with 10^8^ CFU of JKD6159-gD. **k**, **l** The bacterial load in the (**k**) lung was measured on day 5 post *S. aureus* infection. Symbols represent individual mice (*n* = 4–8 mice per group). The result is a combination of three independent experiments (one-way ANOVA, Tukey’s multiple comparison). The dotted line represents the limit of detection. **l** Graph depicts the proportion of mice that were colonised at day 5 post bacterial infection (Fisher’s exact test).

The data in Fig. 3 shows that *S. aureus* reactive CD4^+^ memory T cells facilitates *S*. *aureus* clearance from the lung. To determine whether circulating memory T cells or pulmonary CD4^+^ Trm were responsible for this enhanced clearance of *S. aureus* from the lung tissue, we generated mice with either circulating memory CD4^+^ T cells or circulating and lung Trm CD4^+^ T cells and checked whether these animals were protected against pulmonary *S. aureus* infection. Mice seeded with in vitro activated effector gDT-2 CD4^+^ T cells were immunised intranasally with gD peptide plus adjuvant (LPS), or alternatively, given adjuvant alone and were then rested for 20 days (Fig. 5a). Measuring the absolute number of gDT-2 memory CD4^+^ T cells in the spleen and lung 20 days after the final immunisation showed that while both cohorts had similar numbers of memory gDT-2 CD4^+^ T cells in the spleen, the gD-peptide immunized mice had 8-fold more total gDT-2 CD4^+^ T cells in the lung (Fig. 5b) and 17.5 fold more gDT-2 CD4^+^ Trm in this site compared to the cohort that received adjuvant alone (Fig. 5c). This is in line with previous reports that show lung Trm development is significantly boosted by local cognate antigen recognition^19,37^. Moreover, assessment of the cytokine profile revealed the gD-peptide immunisation also significantly boosted the proportion of IFNγ producing gDT-2 CD4^+^ T cells in the lung tissue (Fig. 5d, e).

We next used this model to assess whether circulating and/or tissue resident *S. aureus* specific memory CD4^+^ T cells could provide protection against severe *S. aureus* pneumonia. Similar to observations in the clinic, we show that prior infection with influenza virus can significantly increase pulmonary *S. aureus* burden and disease severity (Fig S3). To test whether circulating and/or Trm *S. aureus* specific memory CD4^+^ T cells could provide protection against influenza induced *S. aureus* pneumonia, naïve mice, and mice seeded with effector gDT-2 CD45.1^+^ CD4^+^ T cells and intranasally immunised as described above with gD peptide and adjuvant (Act. gDT-2 + gD peptide), or adjuvant alone (Act. gDT-2) were infected intranasally with 10^4^ PFU of influenza A virus (X31, H3N2) and 2 days later, were intranasally infected with JKD6159-gD. Assessment of lung tissue sections 2 days following *S. aureus* infection showed that in comparison to the other cohorts, animals with boosted lung Trm (Act. gDT-2 + gD peptide) had larger numbers of CD3^+^ cells and fewer *S. aureus* bacterium present in their lung tissue sections (Fig. 5f, g). Both naïve mice and the Act. gDT-2 cohort displayed very similar weight loss curves, with all animals losing ~20% of their original body weight, which they did not recovery for the duration of the experiment (Fig. 5h). In contrast, mice with boosted numbers of lung gDT-2 Trm (Act. gDT-2 + gD peptide) lost on average only 10% of their original body weight, and by day 7 p.i. had returned to their pre-infection weights.

Assessment of the bacterial load in the lung on day 5 post *S. aureus* challenge revealed that both the naïve and Act. gDT-2 cohort harboured similar bacterial loads and lung colonisation frequencies (85% and 82%, respectively) (Fig. 5I, j). In contrast, mice with elevated lung gDT-2 Trm displayed lower bacterial loads in the lung (350-fold relative to naïve cohort) and at day 5 p.i. only 25% of animals in this cohort remained colonised (Fig. 5I, j). Collectively, these data show that boosting bacterium specific CD4^+^ Trm along the respiratory tract can significantly reduce the severity of *S. aureus* pneumonia. Moreover, the lack of protection observed in the Act. gDT-2 cohort implies that circulating *S. aureus* specific memory CD4^+^ T cells in the absence of an elevated lung CD4^+^ Trm compartment, has limited capacity to attenuate severe pulmonary *S. aureus* disease. The assessment of the immune cell influx into the respiratory tract 1 day following the bacterial challenge revealed that mice with elevated lung gDT-2 CD4^+^ Trm had a larger pool of neutrophils within the airways in comparison to the non-immunised cohort (Fig S4a). Neutrophils are essential for *S. aureus* clearance, and CD4^+^ T cells support neutrophil activation by secreting IL-17, which recruits neutrophils to the site of infection, and IFN-γ, which can boost neutrophil effector functions. We next assessed whether the protection mediated by bacterium specific CD4^+^ Trm was due to their capacity to boost neutrophil recruitment into the airways. Naïve mice and Act. gDT-2 + gD peptide immunised mice were infected intranasally with 10^4^ PFU of influenza A virus (X31, H3N2) and 2 days later were intranasally infected with JKD6159-gD and administered either anti-Ly6g (1A8) daily to deplete neutrophils, or as a control, given saline (Fig S4b–d). Assessment of the bacterial load in the lung on day 3 post *S. aureus* challenge revealed that mice with elevated lung gDT-2 CD4^+^ Trm had a lower bacterial burden compared to unimmunised mice. While neutrophil depletion throughout the bacterial infection resulted in a 10-fold increase in lung bacterial loads within this cohort, this titre was still significantly lower (100-fold) than that observed in the lung of non-immunised neutrophil depleted controls (Fig S4d). These results imply that the protection mediated by bacterium specific lung gDT-2 CD4^+^ Trm is not solely reliant on their capacity to boost neutrophil recruitment. Interestingly, the protection mediated by the lung gDT-2 CD4^+^ Trm that we observed was dependent on the capacity of these cells to synthesize IFNγ. This was shown through repeating experiments described above, but instead using IFNγ deficient gDT-2, which convert into lung Trm as effectively as their wildtype counterparts and were predominately Th17 polarised (Fig S5a–e). Here we failed to observe any protection by these locally deposited bacterium specific Trm (Fig. 5k, l). Overall, our findings highlight the potential of targeting lung CD4^+^ Th1 polarised Trm in vaccines against invasive pulmonary *S. aureus* infections.

### *S. aureus* specific CD4^+^ memory Th1 cells are present at low frequencies in human lung tissue

**Fig. 6:**
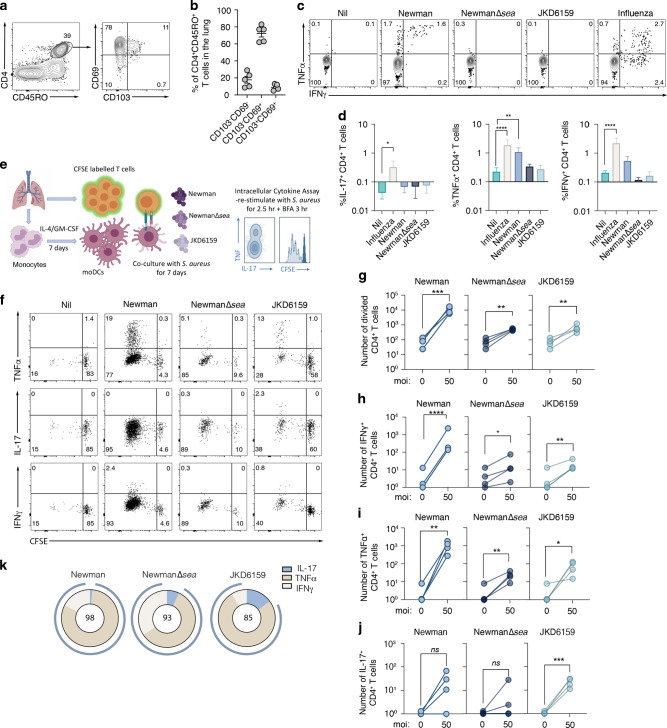
*S. aureus* reactive CD4^+^ Trm are present in human lung tissue. **a** Frequency of CD4 Trm in human lung. **a** Flow cytometry profiles depicting the level of expression of CD103 and CD69 on memory CD4 (CD45RO^+^CD4^+^CD3^+^) T cells isolated from human lung. **b** The percentages of antigen experienced (CD45RO^+^) CD4^+^ T cells in human lung tissue that express CD103 and CD69. Dots represent individual donors (*n* = 5 healthy lungs), and bars show mean ± SEM. **c**–**k** S. aureus specific CD4^*+*^ T cells are present in human lung and are largely Th1 polarised. **c** Monocyte derived dendritic cells (moDCs) differentiated from monocytes isolated from human lung tissue were cultured 1:1 with purified autologous T cells and either heat killed *S. aureus* (moi 50) or influenza A virus (H1N1pdm09, moi 10) for 18 h and the proportion of CD4^+^ T cells producing IFNγ, TNF or IL-17 was measured by intracellular cytokine staining. **c** Flow cytometry profiles depicting the production of IFNγ and TNF in CD4^+^ T cells. **d** The percentage of CD4^+^ T cells synthesizing IL-17, TNF or IFNγ. Bars represent the mean ± SEM. Data pooled from 6 donors (two-way ANOVA, Dunnett’s multiple comparison). **e**–**k** Lung derived moDCs were cultured 1:1 with purified autologous CFSE labelled T cells and heat killed *S. aureus* (moi 50) for 7 days. Following a brief re-stimulation with heat killed *S. aureus*, the proportion of T cells producing IFNγ, TNF or IL-17 was measured by intracellular cytokine staining. **e** Schematic representation of the in vitro *S. aureus* human lung co-culture assay. **f** Representative flow cytometry profiles gated on CD4^+^ T cells. **g** The absolute number of divided CD4^+^ T cells on day 7 post culture (**h**–**j**) The absolute number of divided CD4^+^ T cells synthesizing (**h**) IFNγ (**i**) TNF (**j**) IL-17 on day 7 post culture following a brief in vitro restimulation. Symbols represent individual donors. **k** Pie graphs show the proportion of total cytokine producing cells that are synthesizing IFNγ, TNF or IL-17. Data pooled from four donors. Numbers inside the plots represent and blue outer circle shows the proportion of Th1 producing cells.

In the present study, we show *S. aureus* reactive Th1 skewed Trm in the lungs of mice can reduce the severity of *S. aureus* pneumonia. While a high frequency of *S. aureus*-reactive memory CD4^+^ T cell has been reported in the blood^10,38^ and skin^39^ of healthy patients it is unclear whether these cells also reside in the lung or whether they adopt the required Th1 polarity needed for enhanced bacterial clearance. We next set out to develop an assay to measure the frequency and polarity of *S. aureus* reactive CD4^+^ Trm cells in human lung tissue. Many strains of *S. aureus* produce superantigens (sAgs) that non-specifically activate T-cells, resulting in polyclonal T cell activation and cytokine release^40^. To avoid sAg interference in our quest to identify *S. aureus* specific CD4^+^ T cells, we selected, as a source of antigen, the JKD6159 strain, as it encodes no known superantigens and is the dominant clone of CA-MRSA in Australia,^41^ and strain Newman, as it produces only one sAg, Staphylococcal enterotoxin A (SEA), which we deleted (Newman Δ*sea*)^42^. We validated that these strains were unable to promote non-specific hyperactivation of polyclonal T cells (Fig S6a–c), and then used them in a co-culture assay to measure the frequency and polarity of *S. aureus* reactive CD4^+^ Trm cells in human lung tissue. We and others have previously reported that the human lung harbours a large pool of CD4^+^ Trm (>70% CD45RO^+^CD4^+^ T cells in human lung tissue express the Trm marker CD69)^43–45^ (Fig. 6a, b). To measure the proportion of CD4^+^ Trm in the human lung that were reactive to *S. aureus*, monocyte derived dendritic cells (moDCs) generated from monocytes purified from lung tissue, were cultured with autologous lung T cells and heat killed *S. aureus* (JKD6159, Newman Δ*sea* or as a control, the parental Newman strain) in the presence of brefeldin A. The proportion of CD4^+^ T cells synthesizing TNF, IFNγ or IL-17 were measured by intracellular cytokine staining 18 h later. While exposure to the parental, sAg^+^ Newman *S. aureus* strain triggered lung CD4^+^ memory T cells to non-specifically produce Th1 cytokines (TNF and/or IFNγ), neither of the sAg deficient *S. aureus* strains resulted in CD4^+^ T cytokine production above the background levels observed in cultures without bacteria (Fig. 6c, d). These observations imply that the frequency of *S. aureus* reactive CD4^+^ T cells in human lung is below the detection limit of this assay, which we show, does have the range to detect CD4^+^ T cells specific for other respiratory pathogens, such as influenza virus (Fig. 6c, d).

As the frequency of *S. aureus* reactive T cells in human lung tissue was too low to directly measure ex vivo, we modified our protocol to expand up *S. aureus* reactive cells from lung tissue. To do this, lung derived moDCs were exposed to heat killed *S. aureus* strains and CFSE labelled matched T cells for 7 days. Following a brief restimulation with heat killed bacteria, cytokine production within the expanded T cell pool was measured (Fig. 6e). Once again, exposure of the lung cells to Newman (sAg^+^) resulted in robust non-specific expansion of the bulk CD4^+^ T cell pool (100-fold above cultures without bacteria) and significant TNF and IFNγ production (Fig. 6f–j). Exposure to the sAg^−^ strains did result in ~8-fold expansion of total CD4^+^ T cells and assessment of the polarity of divided (CFSE^lo^) cells revealed that these cells were capable of synthesizing TNF, IFNγ and IL-17 in response to *S. aureus* restimulation (Fig. 6f–j), with most cells adopting a Th1 profile (Fig. 6k). These data suggest that *S. aureus* reactive CD4^+^ memory T cells are present in human lung tissue, albeit at a low frequency, nonetheless following re-exposure to *S. aureus* these lung bacterium specific T cells can expand to generate both Th1 and Th17 populations.

## Discussion

Invasive *S. aureus* infections are a leading cause of morbidity and mortality. The clinical management of these infections is complicated by the presence of widespread antibiotic resistance^46^. To date, all vaccine candidates against *S. aureus*, which largely aim at generating high titres of opsonic antibodies against bacteria surface antigens have failed, and correlates of human immunity remain elusive^47^. For immune-based therapies to provide an alternative to antibiotics in the management of invasive infection, an increased understanding of correlates of protective immunity against *S. aureus* infection is imperative. Here we show that strategies that lodge Th1 polarised bacterium specific CD4^+^ Trm in the lung can significantly attenuate the severity of pulmonary *S. aureus* infections and highlight the potential for targeting these cells in vaccines that aim to prevent the development of *S. aureus* pneumonia.

Trm cells represent a distinct, self-renewing and highly protective population of memory T cells that persist long term in peripheral tissues, commonly at sites of prior infection. Trm cells have been identified in several tissues in mice and humans^13–17,43^ and rapidly acquire effector function after a secondary encounter with a pathogen. Lung CD4^+^ Trm have been shown to be provide long term protective immunity against a range of bacterial infections, including *B. pertussis*^24^, *K. pneumoniae*^25^, *and Streptococcus pneumoniae*^23^. In these infection models, bacterial clearance coincided with the reactivation of Th17 polarised CD4^+^ Trm which accelerated neutrophil recruitment into the airways. While many studies clearly show a role for Th17 polarised CD4^+^ Trm in the protection against extracellular pulmonary bacterial infections, in the present study we find that IFNγ producing Th1 skewed CD4^+^ Trm cells contribute to *S. aureus* defence. Our results are consistent with an elegant study performed by Brown and colleagues that showed the adoptive transfer of in vitro-expanded *S. aureus*-specific Th1 but not Th17 cells conferred clinical protection against a systemic *S. aureus* infection^10^. The protection mediated by IFNγ producing lung CD4^+^ Trm following bacterial challenge is not due to the ability of these cells to impact neutrophil influx into the airways, hence other modes of action will need to be explored in future studies. One potential mechanism these cells may protect against lung bacterial infections is via boosting alveolar macrophage killing of ingested bacteria. The activation of macrophages can be boosted by IFNγ exposure, which triggers NADPH oxidase activation and augments killing of ingested bacteria^48^. An example where this mechanism of protection is observed has recently been reported in a *Mycobacterium tuberculosis (Mtb)* infection model. Inhalation of *Mtb* results in the deposition of lung Th1 CD4^+^ Trm cells that release IFNγ in response to *Mtb* antigen re-exposure^49^, and this serves to enhances macrophage killing of persisting intracellular *Mtb*. It is well documented that macrophages facilitate the clearance of pulmonary *S. aureus* infections and several murine airway *S. aureus* infection models show macrophage depletion results in significant increases in mortality^50^ and lung bacterial loads^51^. Whether IFNγ synthesized by *S. aureus*-activated lung CD4^+^ Trm cells is facilitating *S. aureus* clearance through enhanced macrophage activation, or via an alternative mechanism is an important question that will need to be addressed in future studies.

By combining an *S. aureus* strain expressing a trackable CD4^+^ T cell epitope and a murine model of *S. aureus* pneumonia, we show that pulmonary *S. aureus* infection triggers the activation of bacterial-specific CD4^+^ Th17 cells, but fails to deposit bacterial-specific CD4^+^ Trm along the respiratory tract. The poor seeding of lung Trm we observe following pulmonary *S. aureus* infection may, in part, be explained by a mismatch in the chemotactic gradient generated in the bacteria infected lung and the expression of homing receptors on *S. aureus* specific effector CD4^+^ T cells. We show that *S. aureus* infection generates effector CD4^+^ T cells that express the chemokine receptor CCR6 but not CXCR3 – a profile consistent with that of Th17 effector T cells. Previous reports show lung CD4^+^ Trm uniformly express the chemokine receptor CXCR3^45^, the expression of which was important for T cell recruitment into inflamed lung tissue^36,52,53^. Moreover, assessment of the levels of expression of a panel of chemokines in lung tissue homogenates revealed that by day 5 post *S. aureus* infection only CXCL9, which elicits its chemotactic function by interacting with CXCR3 was still elevated in the lung tissue. This disparity in chemokine receptor/chemokine gradient may explain the limited recruitment and ultimate retention of effector CD4^+^ T cells in the lung following *S. aureus* infection. In addition, the swift sequestration of *S. aureus* by neutrophils in the airways^31^ which results in the rapid clearance of bacteria from the lung may limit local antigen availability – a key requirement for boosting lung Trm development^21,37^. Indeed, many of the pulmonary bacterial infections (*B. pertussis, Mtb, K. pneumoniae*, and *S. pneumoniae*) that have been documented to cause the deposition of lung CD4^+^ Trm, result in high bacterial burdens within the lung tissue for an extended period. The immunological memory formed following primary *S. aureus* infection provided no protection against a secondary *S. aureus* challenge, implying primary *S. aureus* infection triggers the wrong polarity and/or subset of memory T cell needed for protection. This is consistent with our later findings where we show that circulating memory CD4^+^ T cells have limited capacity to assist in the control of pulmonary *S. aureus* infection and only bacterium specific lung CD4^+^ Th1 polarised Trm can significantly attenuate the severity of *S. aureus* pneumonia.

The high rate of recurring *S. aureus* infections among otherwise healthy individuals who have no known immune deficiencies or risk factors suggests that individuals fail to develop effective protective immunity following natural infection^54^. The inability to develop effective immunity against *S. aureus* may be related to the nature of its lifestyle as a colonising opportunistic pathogen. *S. aureus* asymptomatically colonises the nasal cavity of up to 30% of the human population and generally only causes disease when introduced into a susceptible body site or when hosts are immunologically compromised. Moreover, as *S. aureus* colonizes up to 50% of infants by 8 weeks of age^55^, it is feasible that immunity towards this commensal is programmed early in life. Studies using a murine *Staphylococcus epidermidis* neonatal skin colonization model show that early antigen exposure promotes immunological tolerance and the establishment of commensal specific Tregs^56^. Early exposure to *S. aureus* as a commensal may modulate the establishment of T cell-mediated immunity, adversely affecting long-term protection and impacting an individual’s capacity to combat pathogenic infections by this bacterium. Unlike T cells specific for self-antigens which are purged from the T cell repertoire, commensal specific T cells do not undergo negative selection in the thymus and are present in healthy individuals despite the constant presence of their cognate antigens^57^. While it is important to maintain T cell homeostasis toward commensal antigens, it is also important that these cells can be “called to arms” when a commensal develops into a pathogenic threat.

Colonising opportunistic pathogens like *S. aureus* can acquire or select for resistance to antibiotics, a characteristic driven inadvertently by selective pressure applied by any course of antibiotics taken throughout one’s life. As very few effective bactericidal antibiotic treatment options remain, decolonisation has been proposed as an alternative prophylactic therapy for problematic colonising opportunistic pathogens. Immune-based therapies may be a means to eliminate high-risk opportunistic commensal organisms from the microbiome. Here we show that strategies that lodge Th1 polarised bacterium specific CD4^+^ Trm in the lung can significantly attenuate the severity of *S. aureus* pneumonia. Whether approaches that seed *S. aureus* reactive CD4^+^ Trm in niches that are considered principal habitats for this commensal bacterium (ie skin or nasal mucosa), can drive and maintain *S. aureus* decolonisation requires further investigation.

*S. aureus* reactive CD4^+^ Trm cells are present in human lung tissue and these cells can reactivate following re-exposure to *S. aureus* to synthesize TNF, IL-17 and to a lesser degree IFNγ. Our findings are consistent with work by Brown et al., who show that *S. aureus* specific IFNγ producing CD4^+^ T cells (Th1) are expanded in humans during an *S. aureus* bloodstream infection^10^ and Hendricks et al.^39^, who show that both IFNγ as well as IL-17 producing *S. aureus*-specific CD4^+^ Trm are present in the skin of healthy individuals. While we observe a low frequency of *S. aureus* reactive T cells in human lung tissue, it is estimated that on average, ~3.6% of T-cells (range 0.2%–5.7%) present in human peripheral blood respond to *S. aureus* extracellular antigens^38^. This raises an important question, how can humans who harbour such a high frequency of *S. aureus* specific T cells in their blood be so vulnerable to recurring *S. aureus* disease? A clue may lie in the type of immunity needed to fend off an *S. aureus* infection. While circulating memory CD4^+^ T cells have been shown to be beneficial for controlling systemic blood stream *S. aureus* infections^10^, findings generated in the present study reveal that localised *S. aureus* infections are best combatted by localised immune responses. In humans, the low frequency of *S. aureus* reactive CD4^+^ Trm cells in lung tissue may leave individuals susceptibility to pulmonary *S. aureus* infection, despite high frequencies of circulating *S. aureus* specific T cells in the blood. Thus, a vaccine that aims to protect against localised invasive *S. aureus* infections in the heart (i.e., endocarditis) bone (i.e., osteomyelitis), or lung (i.e., pneumonia) should aim to boost bacterium specific CD4^+^ Th1 polarised Trm in these tissue compartments.

A vaccine that prevents infections caused by *S. aureus*, including drug-resistant strains, will save lives and curb the spread of the bacteria and antibiotic resistance. Our findings reveal the protective capacity *S. aureus* specific respiratory tract CD4^+^ Th1 skewed Trm cells and highlight the potential for targeting these cells in vaccines that aim to prevent the development of *S. aureus* pneumonia. Whether CD4^+^ Trm cells can safeguard other tissues against invasive *S. aureus* infections, and whether these Trm, in addition to responding to *S. aureus* dissemination can also sustain decolonisation, are important questions that will need to be addressed in future studies. The answers may lead to improved vaccines that purge the body of this opportunistic pathogen and in doing so, significantly reduce the risk of invasive *S. aureus* disease.

## Methods

### Ethics statement

All animal experiments were done in accordance with the Institutional Animal Care and Use Committee guidelines of the University of Melbourne and were approved by the University of Melbourne AEC (APP1714249, 2015181). Human experimental work was conducted according to the Declaration of Helsinki Principles and to the Australian National Health and Medical Research Council (NHMRC) Code of Practice. Lung samples from deceased organ donors were obtained via the Alfred Hospital’s Lung Tissue Biobank (Supplementary Table 1). Mononuclear cells were isolated and cryopreserved from lungs as previously described^43,44^. PBMCs were isolated from buffy packs obtained from the Australian Red Cross Life Blood (West Melbourne, Australia). PBMCs were isolated by Ficoll-Paque density-gradient centrifugation and cryopreserved as previously described^43,44^. All experiments were done in accordance with the Institutional Human Ethics Committee guidelines of the University of Melbourne and were approved by the University of Melbourne Human Ethics Committee (13908, 1852417).

### Mice, pathogens, and infections

Female (6–14-week-old) C57BL/6 (CD45.2), gDT-2.CD45.1, gDT-2.CD45.1.IFNγ KO mice were bred in-house and housed in specific pathogen-free conditions in the Biological Research Facility (BRF) at the Doherty Institute for Infection and Immunity, the University of Melbourne. All mouse strains used were shown not to carry *S. aureus*. The gDT-2.CD45.1 CD4 TCR-transgenic mice express a TCR that recognizes the HSV-1 immunodominant determinant gD_315-327_ and carry the allelic marker CD45.1^32^. Mice were infected intranasally with 10^4^ PFU of influenza A virus X31 (H3N2) in a volume of 30 μl. Mice were infected intranasally with 10^8^ CFU of *S. aureus* (JKD6159 or JKD6159-gD) in a volume of 10 (upper respiratory tract infection) or 30 μl (total respiratory tract infection). Mice were infected intranasally with 10^6^ PFU of herpes simplex virus (SC16) in a volume of 30 μl.

### Preparation of *S. aureus* for intranasal inoculation

Streptomycin resistant clones of each *S. aureus* (Newman, Newman Δ*sea* MSSA(CC8); JKD6159, CA-MRSA(ST93)) were grown as previously described^31^.

### Enumeration of *S. aureus* in organs

To evaluate bacterial load, lung and nasal tissue were harvested into 1 ml PBS, homogenized and serial dilutions of organ homogenates were made, plated onto streptomycin containing brain heart infusion (BHI) agar plates, and incubated overnight at 37 °C.

#### Construction of *S. aureus* JKD6159-gD

Polymerase chain reaction was conducted with Phusion hotstart DNA polymerase (NEB) following manufacturers recommendations, with oligonucleotides purchased from IDT (Supplementary Table 2). A hybrid construct containing the P*spa* promoter/signal sequence (SS) of Protein A was fused to the serine/aspartic acid (SD) repeats/Sortase A motif of ClfA. The DNA construct was assembled by SOE-PCR with primers IM860/IMT1; IMT2/IM861 with Newman genomic DNA as template. The product was digested with NcoI/PstI and ligated into complementary digested pIME85 (erythromycin derivative of pIMC85 with the integrase deleted^58^) yielding pIME85(Pspa-SD). The plasmid was propagated in *Escherichia coli* strain DC10B-R (EC10B^59^ with the *dcm* gene deleted). To insert gD_315-327_ epitope from HSV into pIME85(P*spa*-SD), the P*spa*-SS was amplified from Newman genomic DNA with IM860 and the reverse primer IM1315 incorporating the gD epitope. The amplimers were digested with NcoI/SacI and ligated into complementary digested pIME85(Pspa-SD), yielding pIME85(P*spa*-gD-SD). The plasmid was co-electroporated^60^ with the phage integrase expressing pUCɸ85 (Ampicillin/Chloramphenicol marked pUC18 with ɸ85 integrase expressed from Phelp promoter and isolated from IM08B) into JKD6159Δ*hsdR* Δ*hsdR*^SCC^^61^ and plated onto BHI agar containing 10 μg/ml erythromycin. Transient expression of the integrase in the presence of the AttP containing pIME85 leads to single copy integration of pIME85(P*spa*-gD-SD) between *rpmF* and *isdB* gene. The stably integrated plasmid was then transduced^62^ into JKD6159^STR^ with phage 11 yielding JKD6159-gD. The resulting strain was whole genome sequenced with Illumina short reads, which were assembled to the JKD6159 reference genome in Geneious Prime. No SNP or indels were identified and single copy integration was confirmed at the correct location.

#### Construction of SEA deficient *S. aureus*

A deletion construct was amplified by SOE-PCR with primers IM1443/IM1444; IM1445/IM1446, (Supplementary Table 2) the gel extracted AD product cloned into pIMAY-Z by SLiCE and transformed into *E. coli* IM08B. The *sea* gene was deleted from Newman^STR^ by allelic exchange^63^. The Newman^STR^Δ*sea* mutant was whole genome sequenced with Illumina short reads, which were assembled to the Newman UoM^64^ reference genome in Geneious Prime. No SNP or indels were identified.

### Cytometric bead array

Cytokine/chemokine concentrations in supernatants from homogenized lung tissue was measured using a LegendPlex mouse antiviral response cytometric bead array following manufacturer’s instructions.

### Real time PCR

Single cell suspensions of lung tissue were washed twice with PBS before RNA extraction by RNeasy Plus Mini Kit (QIAGEN, Venlo, Netherlands). RNA template was prepared, at equal concentrations, and treated to in-solution deoxyribonuclease I digestion (Sigma-Aldrich, St. Louis, USA). Equal volumes of RNA template were used for each SensiFAST cDNA (complementary DNA) synthesis reaction (Bioline, London, UK) before resuspension of template in equal volumes of ultrapure high-performance liquid chromatography water. Real-time polymerase chain reaction was performed with SensiFAST Lo-ROX SYBR Green (Bioline, London, UK) on Mx3005P (Stratagene, La Jolla, USA). Fold change was calculated relative to the geometric mean of housekeeping gene (GAPDH).

### Flow cytometry and intracellular cytokine staining

Mice were perfused before harvest of the lung and nasal tissue. The nasal tissue, including the nasal cavity, nasal turbinates, and NALTs, was obtained by cutting down the vertical plane of the skull and scraping out the tissues and small bones from both sides of the nasal passages. Lung and nasal tissue were enzymatically digested for 1 h at 37 °C in 3 ml of collagenase type 3 (3 mg ml^−1^ in RPMI-1640 supplemented with 2% fetal calf serum). Single cell suspensions were surface stained for 25 min on ice with the appropriate mixture of mAbs. The conjugated mAbs were obtained from BioLegend, or Miltenyi Biotec (San Diego, CA) and are listed in Supplementary Table 3. For intracellular cytokine staining, single cells suspensions were incubated for 1 h with either 10^−6^M gD_315-327_ (IPPNWHIPSIQDA) peptide or PMA/ION and then cultured for an additional 5 h with Golgi plug (Brefeldin A) at 37 °C in complete RPMI (10% FCS, 2 mM glutamine, 50 mM 2-ME, Pen/Strep) prior to surface staining for 25 min on ice with the appropriate mixture of mAbs and then intracellular staining using a Foxp3 fix/perm kit (ThermoFisher). Samples were acquired using a Becton Dickinson Fortessa III flow cytometer and data analysed using the FlowJo software package (Tree Star, Inc., Ashland, OR, USA).

### Purification and adoptive transfer of T cells

Naïve gDT-2 CD45.1^+^ CD4^+^ T cells isolated from gDT-2 TCR transgenics mice were purified from single cell suspensions prepared from LN and spleen. Cells were purified after a depletion step using rat antibodies against CD11b (M1/70), F4/80, Ter-119, Gr-1 (RB6), MHC class II (M5/114), and CD8 (53-6.7), followed by incubation with anti-rat IgG-coupled magnetic beads (BioMags, Qiagen) following the manufacturer’s protocols. Mice received either 50,000 or 1 × 10^6^ CFSE labelled gDT-2 CD45.1 cells intravenously in a volume of 200 μl.

### In vitro activation and transfer of effector gDT-2 cells

gDT-2 CD4^+^ T cells were activated in vitro with 10^−6^M gD_315-327_ (IPPNWHIPSIQDA) peptide pulsed splenocytes as previously described^17^. This protocol resulted in >90% of cells adopting a Th1 polarity.

Mice were injected intravenously with 5 × 10^6^ in effectors and at day 0 and 7 post transfer mice were administered intranasally 1 μg lipopolysaccharide with or without 30 μg of gD_315-327_ peptide in a volume of 30 μl.

### Immunohistochemistry

Perfused lung tissue, inflated with OCT were immediately embedded in OCT. Frozen sections (14 μm) were cut using a cryostat. Tissue sections were fixed with acetone and blocked in serum-free protein block (DAKO, Santa Clara, CA). Sections were stained with a polyclonal rabbit anti*-S. aureus* antibody (ThermoFisher, Cat#PA1-7246), anti-mouse CD3(17A2)-Alexa647 and DAPI. The coverslips were mounted in Prolong Gold Antifade medium. Slides were imaged using a Zeiss LSM700 microscope and images were analysed using ImageJ (version 2).

### Antigen presentation assay

*Dendritic cell isolation*: As previously described^33^, spleen or cervical (cLN) and mediastinal (mLN) lymph nodes recovered from five mice (pooled) were digested with DNAse (Roche) and collagenase (Worthington type 3) to generate single-cell suspensions. Light-density cells were selected using Nycodenz (Nycomed Pharma) (1.077 g/cm^3^). DCs were further enriched by depletion of unwanted cells with rat antibodies directed against CD3 (clone KT3-1.1), Thy1 (clone T24/31.7), CD45R (clone RA36B2), Ly6C/G (clone RB6-8C5), Ly-76 (Ter119) and anti-rat Ig magnetic beads (BioMags, Qiagen). All DC subsets were then sort purified using a BD Aria III (BD Biosciences) into XCR1^+^ and CD11b^+^ subsets.

In vitro: 5 × 10^4^ CFSE-labelled gDT-2 CD45.1^+^ CD4^+^ T cells were cultured with 1 × 10^4^ sort purified splenic DCs in the presence of 1 μg/ml lipopolysaccharides (LPS) from *Escherichia coli* (Sigma) with varying doses of heat killed *S. aureus* (JKD6169 or JKD6159-gD) in complete media (RPMI 1640 media supplemented with 10% FCS, 50 μM 2-mercaptoethanol, 2 mM L-glutamine, 100 U/ml penicillin, 100 μg/ml streptomycin). T cell proliferation was assessed 60 h later.

Ex vivo: 5 × 10^4^ CFSE-labelled gDT-2 CD45.1^+^ CD4^+^ T cells were cultured 1 × 10^4^ sort purified cLN or mLN CD11b^+^ or XCR1^+^ DCs recovered from mice on days 1–4 post intranasal infection with JKD6159-gD, in complete media (RPMI 1640 media supplemented with 10% FCS, 50 μM 2-mercaptoethanol, 2 mM L-glutamine, 100 U/ml penicillin, 100 μg/ml streptomycin). T cell proliferation was assessed 60 h later.

### PBMC/Human lung *S. aureus* stimulation assay

Blood: Monocyte-derived dendritic cells were generated from PBMCs by incubating CD14-enriched monocytes (MojoSort Human CD14^+^ monocyte isolation kit, Biolegend) for 5 days in RPMI 1640 media supplemented with 10% FCS and 300 U/ml hGM-CSF (Peprotech) and 100 U/ml hIL-4 (Peprotech). moDCs were harvested and 2.5 × 10^4^ cells were co-cultured with 5 × 10^4^ matched CFSE labelled T cells and the indicated dose of heat killed *S. aureus*. The absolute number of divided cells (CFSE^lo^) was measured following 7 days of culture.

Lung: Monocyte-derived dendritic cells were generated from lung by incubating plastic-adherent lung cells for 5 days in RPMI 1640 media supplemented with 10% FCS and 300 U/ml hGM-CSF (Peprotech) and 100 U/ml hIL-4 (Peprotech). moDCs were harvested and co-cultured 1:1 with matched T cells and the indicated dose of heat killed *S. aureus* or influenza A virus (H1N1pdm09) for 3 h in RPMI with 10% fetal calf serum at which point Brefeldin A (BD GolgiPlug) was added and cells were incubated for a further 18 h and cells were intracellularly stained for cytokine production. In some experiments, lung moDCs were co-cultured with matched CFSE labelled T cells and the indicated dose of heat killed *S. aureus* for 7 days at which point the cultures we briefly restimulated with heat killed bacteria in the presence of Brefeldin A and the cells were intracellularly stained for cytokine production.

### Statistical analysis

Comparison between two study groups was statistically evaluated by unpaired two-tailed *t* test or Mann–Whitney test. Comparison between more than two groups (single factor) were evaluated using one-way analysis of variance (ANOVA) with Tukey’s or Dunnett’s multiple comparison. Two-way ANOVA with Sidak’s or multiple comparison on log10-transformed values was used to evaluate more than two groups at different time points. In all tests, statistical significance was quantified as *P < 0.05, **P < 0.01, ***P < 0.001, and ****P < 0.0001. Statistical analysis was performed using GraphPad Prism 8 software.

## Supplementary information

Supplementary information
