# Supplementary material for: Staphylococcus aureus specific lung resident memory CD4+ Th1 cells attenuate the severity of influenza virus induced secondary bacterial pneumonia
Source: Mucosal Immunol. 2022 May 30;15(4):783–96. doi: 10.1038/s41385-022-00529-4 (PMC9148937; doi:10.1038/s41385-022-00529-4)
Supplement: Supplementary file 1 — Supplementary information [file 41385_2022_529_MOESM1_ESM.pdf]

Supplementary Figures:

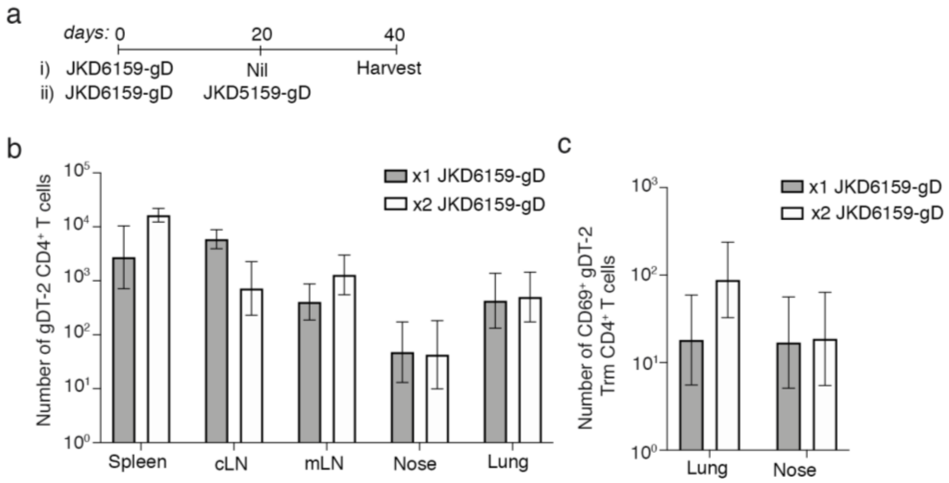

Supplementary Figure 1: Secondary infection does not improve the size of the *S. aureus* specific memory CD4$^+$ T cell response

C57BL/6 (CD45.2) mice injected with 5x10^4 naïve gDT-2 CD45.1$^+$ CD4$^+$ T cells and that received an intranasal (TRT) infection with 10^8 CFU JKD6159-gD were left untreated or re-infected intranasally (TRT) 20 days later with 10^8 CFU JKD6159-gD. (a) Schematic of the experimental timeline. (b-c) Forty days after the primary infection the absolute number of total gDT-2 CD4$^+$ T cells in the spleen, cLN, mLN lung and nose and (c) the absolute number of gDT-2 CD4$^+$ Trm in the lung and nose was measured. Bars represent the mean + SEM (n=6 mice per time point). The result is a combination of 2 independent experiments.

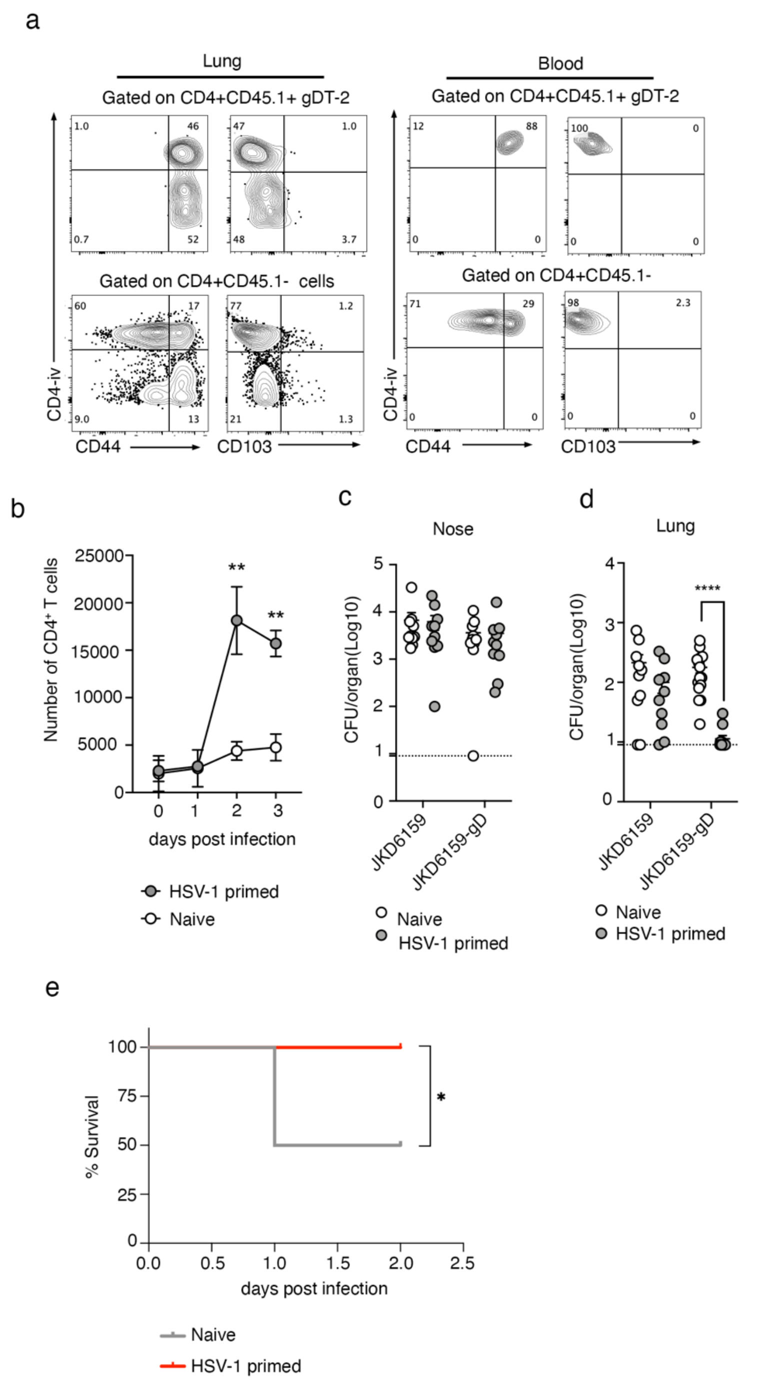

Supplementary Figure 2: Antigen specific protection following *S. aureus* infection of HSV-1 primed mice

(a) C57BL/6 (CD45.2) mice injected with 5x10^4 naïve gDT-2 CD45.1$^+$ CD4$^+$ T cells and that received an intranasal (TRT) infection with 10^6 PFU HSV-1 (SC16) 21 days prior, received 2μg of anti-CD4-PE intravenously just prior to harvest. Flow cytometry analysis on single cell suspensions from lung and blood gated on gDT-2 CD4$^+$ T cells (CD4$^+$CD45.1$^+$) or bulk endogenous CD4$^+$ T cells (CD4$^+$ CD45.1$^-$) showing location (CD8-iv^{lo}: parenchyma associated cells; CD8-iv^{hi} : circulating cells) and the level of expression of CD44 and CD103. (b) Naïve C57BL/6 mice (Naive) or C57BL/6 mice infected intranasally (TRT) 21 days earlier with 10^6 PFU of HSV-1 (HSV-1 primed) were infected intranasally (TRT) with 10^8 CFU of JKD6159-gD and at day 1-3 post infection the absolute number of CD4$^+$ T cells in the bronchial alveolar lavage fluid (BALf) was measured. Symbols represent the mean ± SEM mice (n=6-10 mice per timepoint).

The result is a combination of 3 independent experiments (two-way ANOVA, Sidak's multiple comparison). (c-d) Naïve C57BL/6 mice or C57BL/6 mice infected intranasally (TRT) 21 days earlier with 10^6 PFU of HSV-1 were infected intranasally (TRT) with either 10^8 CFU of JKD6159-gD or the parental strain JKD6159 and at day 2 post infection the bacterial load in the (c) nose and (d) lung was measured. Symbols represent individual mice (n=10-15 mice per timepoint) and the bars represent the mean $\pm$ SEM. The result is a combination of 3 independent experiments (student t test). The dotted line represents the limit of detection. (e) Naïve C57BL/6 mice (Naive) or C57BL/6 mice infected intranasally (TRT) 21 days earlier with 10^6 PFU of HSV-1 (HSV-1 primed) were infected intranasally (TRT) with 4 x 10^8 CFU of JKD6159-gD. The percentage survival was monitored. Graphs depict Kaplan–Meier survival curves (*n*=8–10 mice per group). Data are pooled from 2 independent experiments (Log-rank, Mantel–Cox).

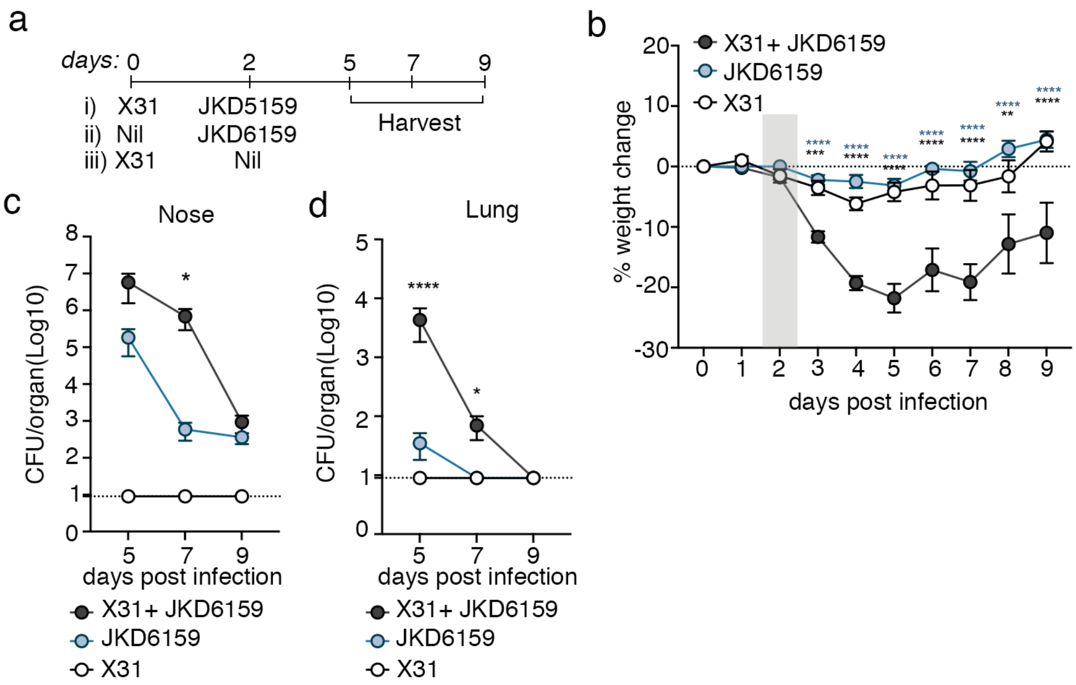

Supplementary Figure 3: Sequential infections with influenza virus followed by *S. aureus* results in the development of severe bacterial pneumonia.

C57BL/6 mice intranasally infected (TRT) with or 10^4 PFU influenza virus (X31, H3N2) and two days later, were infected intranasally (TRT) with 10^8 CFU of *S. aureus* (JKD6159). As controls, mice were infected with either 10^4 PFU influenza virus (X31, H3N2) or 10^8 CFU of *S. aureus* (JKD6159) alone. (a) Schematic of the experimental timeline. (b) The graph depicts the percentage weight change measured over the course of the experiment. The symbols represent the mean $\pm$ SEM (n=12 mice per group). The grey band indicates the day *S. aureus* was administered. The result is a combination of 2 independent experiments (two-way ANOVA, Dunnett's multiple comparison) (c-d) The bacterial load in the (c) nose and (d) lung were measured at the indicated time points. Symbols represent the mean $\pm$ SEM (n=4-7 mice per group per timepoint). The result is a combination of 2 independent experiments (two-way ANOVA, Sidak's multiple comparison). The dotted line represents the limit of detection.

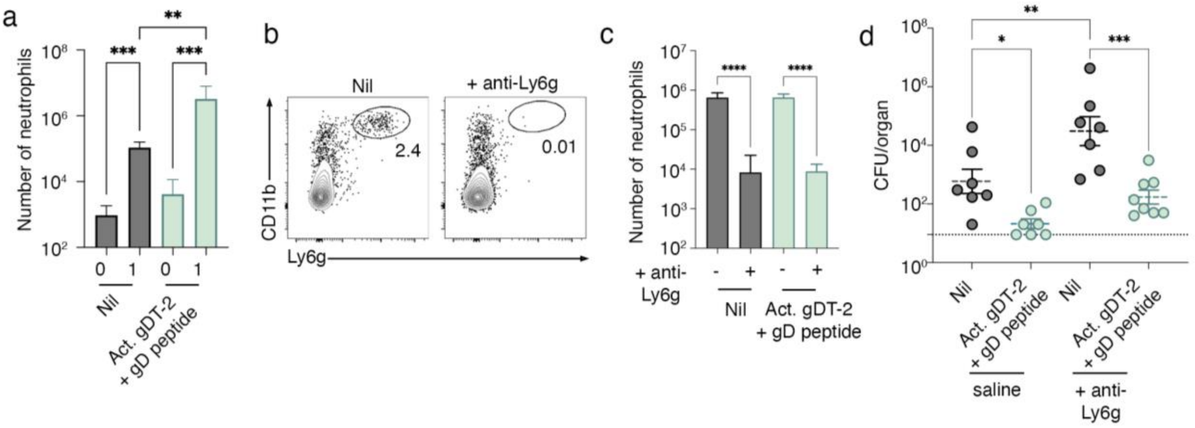

Supplementary Figure 4: The protection mediated by bacterium specific lung gDT-2 CD4^{+} Trm is not abrogated following neutrophil depletion.

(a) C57BL/6 (CD45.2) mice injected with 5x10^{6} *in vitro* activated gDT-2 CD45.1^{+} CD4^{+} T cells were inoculated at day 0 and day 7 via the intranasal route with 1µg LPS with or without 30µg gD peptide. At day 28 post immunisation these mice, and a naïve cohort (Nil) were intranasally infected (TRT) with 10^{4} PFU influenza virus (X31, H3N2) and two days later, were infected intranasally (TRT) with 10^{8} CFU of JKD6159-gD. The absolute number of neutrophils in the airways was quantitated one day later. Bars represent the mean ± SEM (n= 5-7 mice per group). The results are a combination of 2 independent experiments (one-way ANOVA, Tukey's multiple comparison) (b-c) Mice generated as described in **a** were treated with either saline or 250 µg of anti-Ly6g (1A8) daily from the start of the bacterial infection. (b) Representative flow cytometry profiles showing the proportion of neutrophils (CD11b^{+}Ly6g^{+}) cells in the spleen on day 3 post *S. aureus* infection. (c) The absolute number of neutrophils in the spleen on day 3 post *S. aureus* infection. Bars represent the mean ± SEM (n= 4-5 mice per group). The results are a combination of 2 independent experiments (one-way ANOVA, Sidak's multiple comparison). (d) The bacterial load in the lung was measured on day 3 post *S. aureus* infection. Symbols represent individual mice (n=7-8 mice per group). The result is a combination of 3 independent experiments (one-way ANOVA, Sidak's multiple comparison). The dotted line represents the limit of detection.

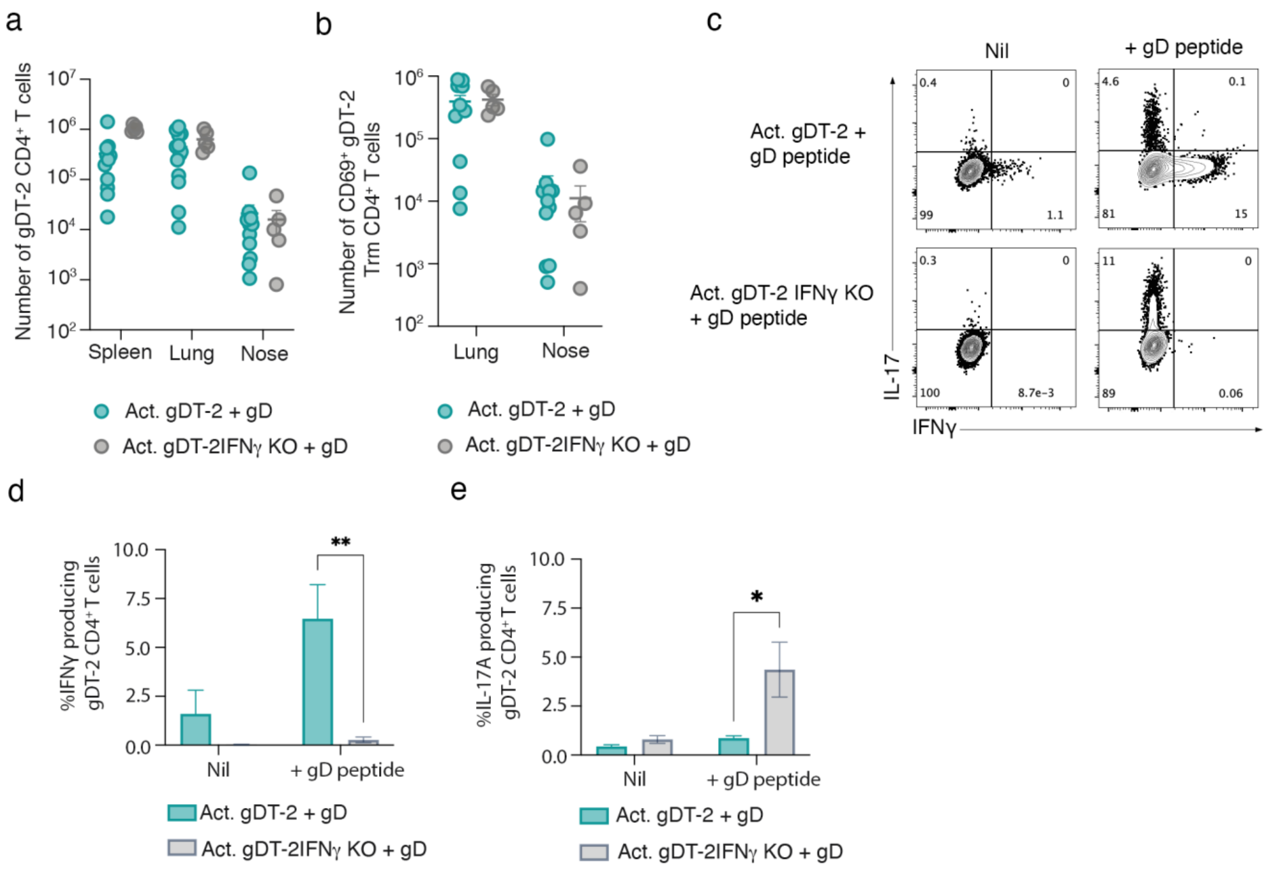

Supplementary Figure 5: IFNγ deficient *in vitro* activated gDT-2 develop into both circulating memory and Trm as effectively as their wild type counterparts and are predominately Th17. C57BL/6 (CD45.2) mice injected with 5 x10^6 *in vitro* activated gDT-2 CD45.1$^+$ CD4$^+$ T cells or gDT-2 CD45.1$^+$IFNγ KO CD4$^+$ T cells were inoculated at day 0 and day 7 via the intranasal route with 1 μg LPS with 30 μg gD peptide, and were rested for 28 days. The (a) absolute number of total gDT-2 CD4$^+$ T cells (CD4$^+$Va3.2$^+$CD45.1$^+$) in the spleen, lung, and nose and (b) the absolute number of gDT-2 CD4$^+$ Trm (CD4$^+$Va3.2$^+$CD45.1$^+$CD69$^+$) in the lung and nose was measured. Symbols represent individual mice, and the line represents the mean ± SEM (n=6-13 mice per group). (c) Representative FACs profiles gated on CD4$^+$ gDT-2 T cells in the lung showing the levels of IFNγ and IL-17 following a brief *in vitro* stimulation. (d-e) The graphs show the proportion of gDT-2 CD4$^+$ T cells in the lung synthesizing (d) IFNγ or (e) IL-17 (n=7 mice per group, two way ANOVA, Sidak's multiple comparison).

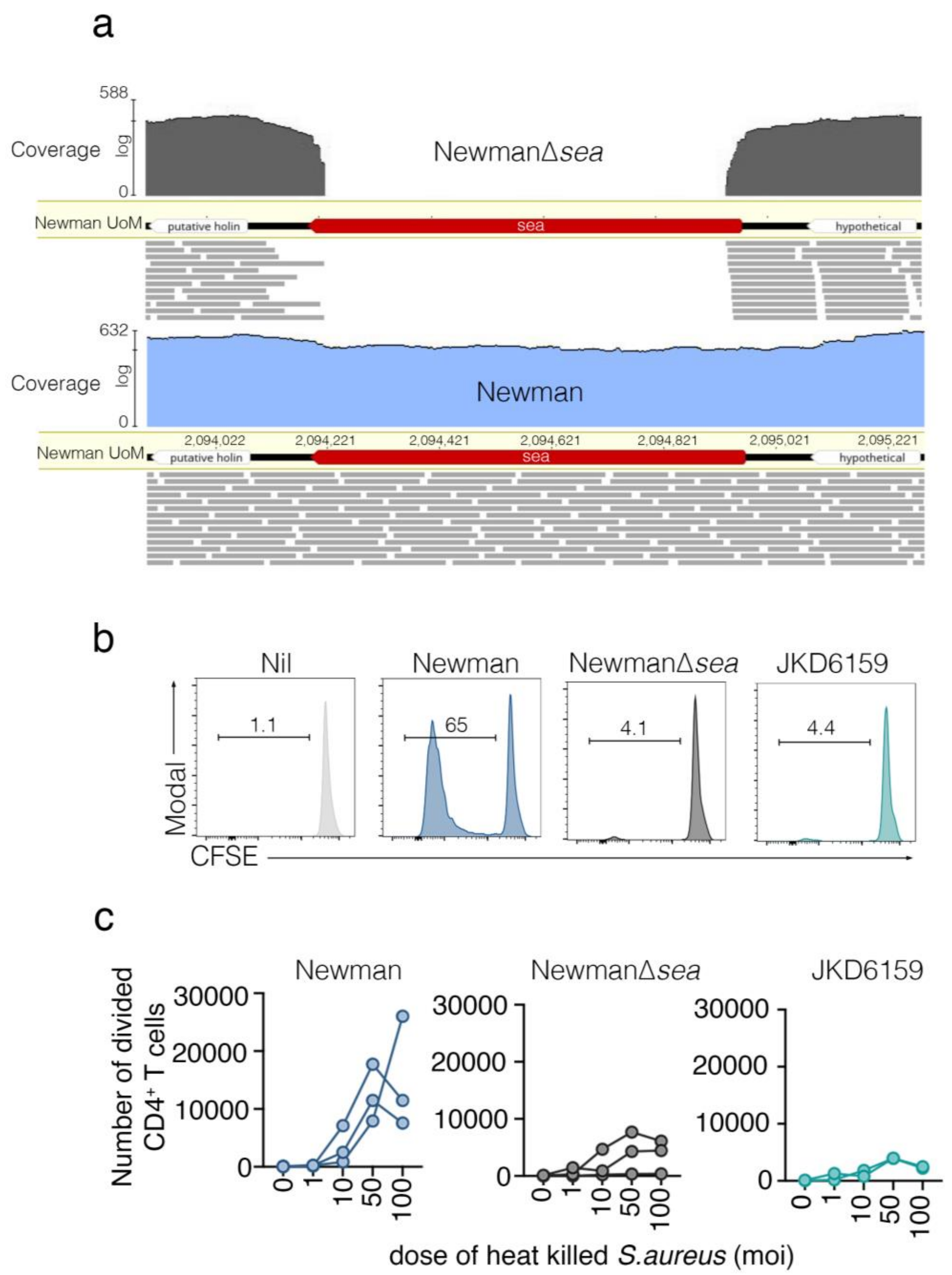

Supplementary Figure 6: The JKD6159 and NewmanΔ*sea* strain of *S. aureus* are devoid of sAgs and do not drive non-specific hyperactivation of CD4$^+$ T cells.

(a) Sequence alignment showing successful deletion of the SEA sAg from Newman strain (b-c) Monocyte derived dendritic cells (moDCs) that we generated from the peripheral blood mononuclear cells (PBMCs) of healthy blood donors were cultured with CFSE labelled matched T cells and varying doses of heat killed JKD6159, NewmanΔ*sea* or as a control, the parental Newman strain, and 7 days later CD4$^+$ T cell expansion was measured. (b) Representative histograms of CFSE dilution of CD4$^+$ T cells following 7 days culture (moi 50). (c) The absolute number of divided CD4$^+$ T cells following 7 days culture. Lines represent individual donors (n=3 donors)

Supplementary table 1: Lung organ donor information

Donor	Age (y)	Sex	HLA-A
**A(19)**	57	F	11,26
**B(17)**	31	M	3.29
**C(10)**	61	M	2, 26
**D(15)**	42	M	2,68
**E(19)**	57	F	11,26
**F (3)**	60	F	29,68
**G(18)**	41	F	2,25
**H(16)**	26	M	1,3
**I(21)**			

Supplementary table 2: Oligonucleotides used in this investigation

ID	Sequence*
IM860	ATATccatggATGACGCGCAGCAAGACC
IMT1	gagctcCGCAGCATTTGCAGCAGGTG
IMT2	CACCTGCTGCAAATGCTGCGgagctcGAACCAATTCCAGAGGATTCAG
IM861	ATATctgcagTTATTTCTTATCTTTATTTTCTTTTTTTCTTCTG
IM1315	ATATgagctcTGCATCCTGAATTGATGGAATATGCCAATTTGGTGGAATCGCAGCATTTGCAGCAGGTGTTAC
IM1443	**CCTCACTAAAGGGAACAAAAGCTGGGTACC**TGATCATGTGCTTGAACTTAGAGAGG
IM1444	CATTTTGCTCACCTCTAAAGCATAATTCC
IM1445	TTATGCTTTAGAGGTGAGCAAAATGTAAACATGGTAGTTTTGAACACGTAATG
IM1446	**CGACTCACTATAGGGCGAATTGGAGCTCC**CCAATAGCTTTTGCGATGTTTTG

***Lower case: Restriction sites; Bold: SLiCE tails.**

Supplementary table 3: Antibodies used in this investigation

Antibody	Clone	Source
Flow cytometry - mouse		
CD8	53-6.7	BioLegend
CD45.1	A20	BioLegend
CD4	GK1.5	BioLegend
CD3	17A2	BioLegend
IL-17	TC11-18H10.1	BioLegend
IFNg	XMG1.2	BioLegend
CD11c	N418	BioLegend
CD11b	M1/70	BioLegend
XCR1	REA7.7	Miltenyi Biotec
CD69	H1-2F3	BioLegend
Va3.2	RR3-16	BioLegend
CXCR3	CXCR3-173	BioLegend
CCR6	29-2L17	BioLegend
CD62L	MEL-14	BioLegend
Flow cytometry - human		
CD4	RPA-TA	BioLegend
CD45RO	UCHL1	ThermoFisher
CD103	Ber-ACT8	BioLegend
CD69	FN50	BioLegend
IFNg	4S.B3	BioLegend
TNF	Mab11	BioLegend
IL-17	BL168	BioLegend
CD3	OKT3	ThermoFisher
